# FEM-Based Estimation–Correction with Minimal Indentation Set for Internal Cavity Classification and Geometry Estimation in Deformable Objects

**DOI:** 10.3390/s26103022

**Published:** 2026-05-11

**Authors:** Thibaut Morant, María Cordero-Alvarado, Tianyi Yang, Koshi Kurosawa, Yuto Tanizaki, Nahoko Nagano, Wenwei Yu

**Affiliations:** 1 Department of Medical Engineering, Chiba University, Chiba 263-8522, Japan; thibaut.morant@chiba-u.jp (T.M.); cgfa0950@chiba-u.jp (M.C.-A.); yang_tianyi_zyzs@chiba-u.jp (T.Y.); 6ko.kuro4@gmail.com (K.K.); 26wd4205@student.gs.chiba-u.jp (Y.T.); 2 Center for Frontier Medical Engineering, Chiba University, Chiba 263-8522, Japan; nagano@chiba-u.jp

**Keywords:** physics-based simulation, internal structure estimation, adaptive grasping, robotic manipulation, robotic sensing

## Abstract

Accurately estimating the internal structure of deformable objects from sparse measurements remains a significant challenge in robotics. This work proposes a three-stage identification framework for this problem. First, a classification strategy determines a minimal informative set of indentation locations using a generalized error computed from pre-simulated FEM force reactions of baseline cavity models and flat-punch indentation estimation. Using this set, the estimation stage detects the cavity type and provides a preliminary estimate of its geometric parameters based solely on measured indentation responses. The correction stage then refines these parameters by replaying measured indentation depths in FEM simulations and deriving geometry corrections from the discrepancy between simulated and homogeneous force responses. Robust loss functions at both stages limit the influence of measurements where local contact conditions deviate from the assumed model, improving reliability across all tested cases. Indentation depth was obtained through gripper proprioception, with an RGB-D camera limited to global pose alignment. Experiments on soft cubes with spherical, cuboid, and pyramidal cavities demonstrate that, within known cavity families and fixed material parameters, the minimal indentation set reliably distinguishes cavity types and the pipeline reconstructs dimensions within error bounds. Extending the framework to non-centered structures and unknown materials remains future work.

## 1. Introduction

In robotics applications such as manipulation, inspection, and safe interaction, estimating an object’s physical parameters remains a fundamental challenge. These parameters include mechanical and material properties such as stiffness, Young’s modulus, Poisson’s ratio, and damping, as well as structural characteristics such as internal geometry with cavities or material inclusions. These parameters govern the object’s response to external forces and are therefore essential for predictive modeling and reliable robotic interaction [1,2,3].

Previous research on deformable-object estimation spans a wide range of assumptions and methodological choices, which can be organized along several methodological axes. Along the modeling axis, methods vary from analytical deformation models to full nonlinear Finite Element Method (FEM) simulations [3,4,5,6]. These methods are typically designed to predict surface deformation or to estimate elasticity parameters under known geometry, and recent developments extend them to interactive or accelerated FEM frameworks for real-time feedback [7,8,9]. However, even these advanced models generally assume that the internal structure is fixed and do not attempt to infer geometric variations such as cavities. Along the sensing axis, systems rely on vision-based reconstruction [10], tactile or force-based probing [6], or combined proprioceptive–exteroceptive strategies [11]. These modalities are effective for recovering global shape or estimating distributed stiffness, but none directly reveal subsurface geometry unless dense measurements or full-field deformation data are available. Along the temporal axis, most inverse elasticity and material-estimation methods rely on offline FEM or commercial FEA solvers, such as Abaqus, ANSYS, or COMSOL [12,13,14], where computation time is not a constraint. Real-time or near-real-time engines, such as SOFA or NiftySim [7,8] and biomechanics-oriented solvers such as FEBio [15], have been primarily used for manipulation, simulation, or control, but not for estimating unknown internal geometry. Moreover, even when accelerated FEM is available, the high dimensionality of cavity shapes makes real-time internal-structure estimation infeasible without additional structural assumptions or sparse probing strategies. Finally, in terms of probing strategy, vision-based observation is sufficient for global deformation tracking, while active probing, such as robotic indentation, improves the identifiability of material parameters [6,16]. Nevertheless, indentation in existing works primarily targets stiffness recovery rather than geometric reconstruction. This is partly because multiple cavity types can produce nearly identical indentation responses unless probing locations are optimized. Across these axes, existing methods demonstrate strong performance for material-parameter estimation or deformation prediction but share a common limitation: they are not designed for internal-structure identification under sparse probing. Furthermore, most methods are ultimately developed to support deformable-object manipulation, where the objective is to enable stable control, grasp planning, or trajectory execution rather than recovering hidden subsurface geometry. Consequently, they are not designed to determine whether a cavity is present, nor its type or dimensions, when only a limited number of indentation measurements are available.

Internal cavities significantly influence load-bearing capacity, deformation behavior, and grasp stability; however, their geometric signatures are spatially concentrated and often weak under sparse probing, making them difficult to identify from surface measurements alone. Prior approaches using FEM-based inverse elasticity [17], elastography-inspired reconstruction [16], three-dimensional inverse elastography [18], or level-set inverse methods for internal geometry recovery [19] typically require dense deformation fields, full-field force information, or high-resolution imaging. These assumptions do not hold in robotic settings where measurements are sparse, and sensing is limited to contact interactions.

To the best of the authors’ knowledge, no existing method directly addresses internal cavity-type classification and parametric geometry estimation from sparse robotic indentation in deformable objects. The closest related works approach the problem from different angles. Inverse FEM with rolling indentation probes has targeted material parameter identification rather than internal geometry, treating geometry as known rather than estimated [20]. Monitored indentation has been used to study force-response sensitivity to inclusion properties in elastomers [21], but without a margin-based selection criterion, cavity-type classification, or parametric geometry estimation.

Robotic palpation with force sensing has been applied to sub-dermal tumor geometry reconstruction [22], but relies on dense contour-following trajectories and does not employ minimal set selection or parametric family classification. FEM-based inverse analysis combined with Bayesian optimization has recovered internal abnormality shape from full-field surface deformation [23], but requires dense imaging input rather than sparse indentation. Sparse probing via elastostatic signed distance functions has been applied to external shape reconstruction without inferring internal void structure [24]. The proposed framework is thus the first to combine margin-based minimal set selection, cavity-type classification, and FEM-based parametric geometry estimation under sparse robotic indentation.

To address these limitations, this work introduces a physics-based framework to determine the minimal set of robotic indentations sufficient to discriminate internal cavity structures under real-world sensing constraints. By leveraging FEM-derived generalized force-responses and a margin-based selection criterion, the proposed method identifies mechanically informative probing locations prior to any real measurement. The approach explicitly separates cavity discrimination, preliminary estimation, and FEM-based correction, ensuring physically consistent geometric refinement at each stage without relying on dense sensing or learning-based models. Experimental validation is conducted to demonstrate the feasibility of reliable cavity-type identification and consistent geometric refinement using a small, fixed number of indentations at selected locations across different gripper configurations. Unlike learning-based tactile perception approaches that require large training datasets and dense sensing, this work focuses on minimizing physical interactions through mechanics-driven selection, without training or prior data collection.

## 2. Methods and Materials

This section describes the method proposed in this study and the materials used to validate it. The proposed approach consists of two components. The first is a theoretical procedure that determines a minimal set of indentation locations capable of discriminating cavity types. The second is an estimation–correction pipeline that reconstructs the internal cavity geometry from sparse experimental measurements and FEM feedback. These two components are then integrated with a robotic indentation setup, a set of fabricated soft objects, and a Neo-Hookean FEM model implemented in SOFA. Finally, the section concludes with the experimental protocol.

### 2.1. Proposed Method

The two components introduced in this work address complementary challenges: selecting where to probe and how to reconstruct internal geometry from the resulting measurements.

The first contribution of this work is a minimal informative set of indentation locations, determined prior to any real experiment from FEM-simulated force responses and flat-punch estimation to maximize discrimination between baseline cavity models. This minimal set serves as the foundation for the entire pipeline, ensuring that the robot performs physical probing only at locations that are theoretically guaranteed to distinguish between internal cavity types. The selection relies on a generalized-error computation that evaluates, for each candidate indentation site, force-response discrepancies across cavity models, with indentation depth contributing as a weighted and normalized consistency term.

The second contribution is an estimation–correction pipeline that reconstructs the cavity type and geometry using sparse real indentation measurements taken exclusively at the locations contained in the minimal set. The estimation stage compares each measured indentation depth against an analytical baseline corresponding to a homogeneous cube to produce an initial cavity classification and preliminary geometric parameters. The correction stage then refines these parameters by simulating the same indentation conditions in SOFA and adjusting the cavity geometry based on discrepancies between simulated and measured reaction forces.

**Figure 1:**
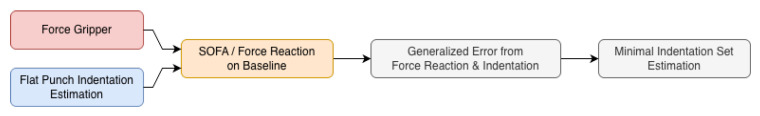
Minimal indentation set identification.

**Figure 2:**
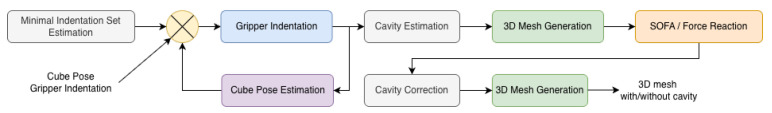
Estimation and correction pipeline: Reconstruction of internal cavities.

Together, these two components constitute a unified approach. The minimal indentation set (stage 1) enables efficient data acquisition (Figure 1), and the estimation–correction pipeline (stage 2 and 3) reconstructs internal cavities using only a small number of measurements (Figure 2). The minimal indentation set is determined prior to the estimation–correction pipeline; both rely on real indentation measurements and FEM force reactions, but also on independent data. The method assumes known cavity families, elastic material behavior, and centered internal structures. These assumptions define the intended scope of the framework rather than incidental limitations: the known-family assumption enables the margin-based discrimination criterion to operate on a fixed hypothesis space, the known-material assumption allows FEM simulations to produce physically consistent force predictions without a preliminary characterization step, and the centered-structure assumption establishes a tractable foundation from which the pipeline can be validated and extended. Each assumption corresponds to a concrete requirement that must be satisfied or verified before applying the pipeline to a new object, and the implications of relaxing them are discussed in Section 4. All hyperparameter values used in the following stages are reported in Table A1 (see Appendix A.5).

The three stages exploit complementary information. The minimal set guarantees that real measurements are mechanically informative by selecting locations where cavity-induced force-response differences exceed a noise-normalized margin. The estimation stage then classifies cavity type and initializes geometry from depth alone, without runtime FEM over the full hypothesis space. The correction stage refines geometry within the selected family using force-response consistency, resolving information that depth measurements alone cannot provide. This separation ensures each stage operates on the most informative signal available without redundant physical interactions or FEM evaluations.

Throughout this work, reconstruction refers to parametric estimation within a fixed hypothesis space, and identification to family selection; neither implies full volumetric recovery.

### 2.2. Method Details

#### 2.2.1. Minimal Indentation Set

The objective of the minimal-set identification stage is to determine a subset of indentation locations that maximizes the discrimination between the cavity models considered in this study. This computation relies on two sources of information. The first one is the reference indentation behavior predicted by an analytical flat-punch approximation for a homogeneous object $h_{\mathrm{flat}(gr)}$ under a given gripper geometry $gr\in \{G_{25},G_{20}\}$ and maximum force $F_{\mathrm{MAX}}$ of the gripper (see Section 2.3). The second one is the reaction forces $F_{\mathrm{SOFA}\_B}\left(m,\ell \right)$ and indentation depths $h_{\mathrm{SOFA}\_B}\left(m,\ell \right)$ obtained from SOFA for a baseline cavity configuration, for each model m∈M and location ℓ∈L. Their discrepancy forms the basis for quantifying the discriminative power of each indentation site, where $\lambda_{h}$ balances the relative contribution of force and depth components:

(1) $$E\left[m,\ell \right]=\sqrt{{\left|\frac{F_{\mathrm{MAX}}-F_{\mathrm{SOFA}\_B}\left(m,\ell \right)}{F_{\mathrm{ref}}}\right|}^{2}+\lambda_{h}^{2}{\left|\frac{h_{\mathrm{flat}(gr)}-h_{\mathrm{SOFA}\_B}\left(m,\ell \right)}{h_{\mathrm{ref}}}\right|}^{2}},$$

where $F_{\mathrm{ref}}=\max\left(\left|F_{\mathrm{SOFA}\_B}\left(m,\ell \right)\right|,\varepsilon_{F}\right)$ and $h_{\mathrm{ref}}=\max\left(\left|h_{\mathrm{SOFA}\_B}\left(m,\ell \right)\right|,\varepsilon_{h}\right)$ are normalization constants based on the magnitude of the SOFA response where $\varepsilon_{F}=0.1$ N and $\varepsilon_{h}=10^{-4}$ mm are small positive floor values, negligible relative to the typical force and depth ranges observed in SOFA simulations. The dispersion of E[m,ℓ] across M quantifies how sensitive location *ℓ* is to changes in internal cavity geometry.

To account for the heterogeneous distribution of locations over the object’s surface, each site *ℓ* is assigned a regional weight:

(2) $$w_{\mathrm{reg}}\left(\ell \right)=\frac{C}{\sqrt{N_{reg(\ell )}}},$$

where reg(ℓ)∈{center,edge,off} denotes its region and $N_{reg(\ell )}$ is the number of candidate locations belonging to that region, and *C* is a normalization constant chosen such that the average weight over all locations equals one. This term ensures that regions with different numbers of candidate locations (see Section 2.3) contribute equally to the discrimination criterion. A second weighting term favors locations whose generalized error varies more strongly across cavity models, thereby promoting sites with higher discriminative potential:

(3) $$w_{\mathrm{diff}}\left(\ell \right)=\min\;\left(\max\left(\frac{s(\ell )}{\mathrm{median}\left(s\right)+\varepsilon_{diff}},0.5\right),2.0\right),$$

where $s\left(\ell \right)={\mathrm{std}}_{m\in M}\left(E\left[m,\ell \right]\right)$ is the standard deviation of the generalized error across the set of cavity models M for a location *ℓ* and where the median is taken over all candidate indentation locations *ℓ*, with $\varepsilon_{diff}>0$, a small numerical stability constant. The final weight assigned to location *ℓ* is given by $w_{loc}\left(\ell \right)=w_{\mathrm{reg}}\left(\ell \right)\cdot w_{\mathrm{diff}}\left(\ell \right)$, normalized, so that its average over L equals one.

The complete procedure for computing the generalized error matrix and the associated location weights is detailed in Algorithm A1 (see Appendix A.7). This construction provides a weighted feature vector for each model at each location, enabling consistent comparison across geometries.

To identify a minimal possible subset of locations, the method evaluates a margin defined as follows for any candidate set S⊆L, where L denotes the full set of candidate indentation locations and each ℓ∈L represents a single location for each model m∈M:

(4) $$\mathrm{margin}\left(S\right)=\frac{\min_{m_{i}\neq m_{j}}{∥v_{m_{i}}\left(S\right)-v_{m_{j}}\left(S\right)∥}_{2}}{\sqrt{\left|S\right|}\;\sigma },$$

denoting the Euclidean distance norm, where:

(5) $$v_{m}\left(S\right)={\left\{w_{loc}\left(\ell \right)\;E\left[m,\ell \right]\right\}}_{\ell \in S,m\in M},$$

is the weighted signature of a model *m* restricted to *S*. A set *S* is considered sufficiently discriminative if margin(S)≥γ. The margin is normalized by $\sqrt{\left|S\right|}\;\sigma$, where |S| is the number of individual indentation locations in *S* and σ is the assumed Gaussian noise level in the normalized error domain, accounting for the increase in noise with set size. In this study, γ=3 enforces a 3σ separation between the closest pair of cavity hypotheses, ensuring robust discriminability under Gaussian noise with σ=0.02.

To structure the search, locations are grouped into candidate sets $G_{\mathrm{cand}}$ (e.g., centers, edges, off-center, axis-aligned pairs). Starting from an empty set, groups are incrementally added until the margin criterion is satisfied. The margin-driven combinatorial search used to identify the optimal minimal subset is formalized in Algorithm A2 (see Appendix A.8).

The resulting set $S^{*}$ defines the minimal collection of indentation locations at which different cavity types produce measurably distinct responses in the generalized-error space. This set is subsequently used in real experiments: all indentation with estimation and correction stages rely exclusively on the locations in $S^{*}$, ensuring that the pipeline remains sparse and efficient. This minimal set is optimal with respect to the considered cavity families, candidate indentation groups, and symmetry assumptions, and does not claim global optimality beyond this constrained design space.

The generalized error and margin are intermediate discriminative measures rather than final decision variables. Their role is to identify the subset of indentation locations that maximizes separability between cavity models before any parameter fitting is performed. This selection step ensures that subsequent cavity estimation is conducted on geometrically informative data rather than on redundant or weakly discriminative indentations.

#### 2.2.2. Estimation Stage

The estimation stage builds upon established inverse indentation methodology. Depth normalization, nonlinear least-squares fitting, robust loss functions, and Bayesian Information Criterion-based (BIC-based) model selection are standard tools in parametric inverse identification [17,25,26,27,28]. The novel component introduced here is the distance-based influence model enabling geometric inference from sparse indentation measurements.

This stage uses real indentation measurements taken only at the locations in $S^{*}$. For each indentation *i*, the robot applies the maximum gripper force and records the resulting depth $d_{{\mathrm{REAL}}_{i}}$. An analytical homogeneous half-space model provides a baseline indentation depth $d_{\mathrm{SOFA}\_B_{i}}$ under identical loading conditions, allowing the computation of a normalized indentation ratio:

(6) $$\rho_{i}=\frac{d_{{\mathrm{REAL}}_{i}}}{d_{\mathrm{SOFA}\_B_{i}}},$$

where $d_{\mathrm{SOFA}\_B_{i}}=h_{\mathrm{SOFA}\_B}\left(m_{full},\ell \right)$ for the homogeneous model $m_{full}$ at location *ℓ* corresponding to indentation *i*. This ratio captures the relative compliance at each indentation site, with values greater than one indicating softer responses (potentially due to cavities).

To relate these ratios to cavity geometry, a distance-based influence model is introduced, which assumes that the compliance perturbation induced by a cavity decays exponentially with the distance from the cavity boundary:

(7) $${\hat{\rho }}_{i}\left(\theta \right)=1+\psi \exp\left(-\frac{d_{\mathrm{out}}\left(p_{i};\theta \right)}{\lambda_{b}}\right),$$

where $p_{i}$ denotes the surface location of indentation *i*, $d_{\mathrm{out}}\left(p_{i};\theta \right)$ is the distance from $p_{i}$ to the nearest cavity boundary defined by parameters θ, ψ is a fitted amplitude parameter, and $\lambda_{b}$ controls how rapidly the cavity influence attenuates with distance. The model recovers ${\hat{\rho }}_{i}\to 1$ as the distance to the cavity boundary increases, consistent with a homogeneous response away from the cavity.

The cavity parameters θ are estimated by minimizing the following energy function:

(8) $$E\left(\theta \right)=\sum_{i}^{k}\omega_{est_{i}}{\left(\rho_{i}-{\hat{\rho }}_{i}\left(\theta \right)\right)}^{2}+\sum_{i,j\in P}^{k,f}\omega_{est_{i,j}}{\left[\left(\rho_{i}-\rho_{j}\right)-\left({\hat{\rho }}_{i}-{\hat{\rho }}_{j}\right)\right]}^{2}+\mathrm{bc}\left(\theta \right),$$

where P denotes pairs of indentations applied on opposite faces of the object. The pointwise weight $\omega_{est_{i}}=0.30$ ensures all selected indentation locations contribute equally to the estimation stage. Its value is chosen so that the pairwise antisymmetry terms remain the dominant contribution to the energy function, and moderate variations around this value are not expected to alter cavity-type selection, consistent with the hyperparameter robustness observed for $\lambda_{b}$. The pairwise weight $\omega_{est_{i,j}}$ is computed adaptively as $\omega_{est_{i,j}}=\min\left(3.0,\;|\Delta_{\mathrm{anti}}|/\sigma_{\mathrm{anti}}\right)$, where $\Delta_{\mathrm{anti}}$ is the measured antisymmetry between opposite-face indentations and $\sigma_{\mathrm{anti}}=0.12$ is a normalization scale. This formulation assigns higher consistency weights to pairs exhibiting stronger antisymmetric responses, up to a cap of 3.0, while avoiding over-penalization of nearly symmetric configurations. The boundary penalty term bc(θ) enforces geometric feasibility of the cavity parameters by discouraging non-physical configurations (e.g., negative dimensions or cavities intersecting the outer surface):

(9) $$\mathrm{bc}\left(\theta \right)=\beta \sum_{i}^{k}{\left(\max(0,\zeta_{i}\left(\theta \right))\right)}^{2},$$

where $\zeta_{i}\left(\theta \right)\leq 0$ are inequality constraints encoding the family-specific geometric conditions and β≥0 is a penalty coefficient chosen sufficiently large to reject infeasible configurations in the cube domain. The cavity parameters θ are used to define the cavity geometry within the selected family:

(10) $$\theta =\left\{\begin{array}{cc} \left(radius\right) & \mathrm{for}\;\mathrm{spherical}\;\mathrm{cavities},\\ \left(s_{h},a_{z}\right) & \mathrm{for}\;\mathrm{cube}\;\mathrm{cavities},\\ \left(a_{x},a_{y},a_{z}\right) & \mathrm{for}\;\mathrm{cuboid}\;\mathrm{cavities},\\ \left(c_{z},a_{x},a_{y},h\right) & \mathrm{for}\;\mathrm{pyramidal}\;\mathrm{cavities}. \end{array}\right.$$

where $a_{x}$, $a_{y}$, $a_{z}$ are half-lengths along each axis, $s_{h}$ is the square base half-length with $a_{x}=a_{y}=s_{h}$, and *h* is the pyramid height. All cavity origins are fixed at (0,0,0) except for the pyramidal cavity, where $c_{z}$ denotes the displacement of the cavity origin along the height axis $A_{o}\in \left\{X,Y,Z\right\}$, which accounts for the asymmetric placement of the pyramid base. The base alignment axis $A_{o}$ is determined by testing each candidate orientation separately rather than by numerical optimization. To improve robustness to outliers during parameter estimation, E(θ) is minimized using a Huber-loss least-squares solver (scipy.optimize.least_squares with loss = “huber” and f_scale = $\delta_{e}=0.15$) [29], which applies an effective per-point weight $\phi_{\delta_{e},i}=\min(1,\delta_{e}/|r_{i}\left|\right)$ to each residual $r_{i}=\rho_{i}-{\hat{\rho }}_{i}\left(\theta \right)$: points with $|r_{i}|\leq \delta_{e}$ contribute fully, while those with $|r_{i}|>\delta_{e}$ are downweighted in proportion to their residual magnitude. As a result, the converged estimate ${\hat{\theta }}_{m}=\arg\min_{\theta }E_{m}\left(\theta \right)$ is driven primarily by indentation points where the distance-based model fits reliably, and is protected from being distorted by points where the force–geometry proportionality assumption locally breaks down.

This breakdown occurs in two distinct regimes. The first is structural thinning: when the cavity wall is thin relative to the contact area and the cavity boundary is geometrically sharp, local deformation becomes strongly nonlinear and the exponential distance-based model cannot capture it, producing large residuals $r_{i}$ and correspondingly small $\phi_{\delta_{e},i}$ [30]. The second is directional geometry mismatch: when the cavity geometry produces compliance variations that the isotropic distance-based exponential model can only partially capture, $\phi_{\delta_{e},i}$ varies across axes rather than being uniformly reduced [31]. In both cases, the Huber-weighted fitting acts as a dynamic reliability filter: measurements producing large residuals are downweighted, and parameter recovery is driven by the more stable, proportionate measurements in the minimal indentation set. The physical manifestation of both regimes under the experimental conditions of this study is illustrated and discussed in detail in Section 3.3.

Once ${\hat{\theta }}_{m}$ is obtained for each cavity family m∈M, ${\mathrm{RSS}}_{m}$ is evaluated at ${\hat{\theta }}_{m}$ as an unweighted sum (Equation (12)), reflecting how well the converged geometry explains all measurements. The BIC then combines ${\mathrm{RSS}}_{m}$ with a complexity penalty (Equation (11)) to select the cavity family that best balances fit quality and model complexity. The Huber loss therefore shapes the BIC selection indirectly: by steering ${\hat{\theta }}_{m}$ toward a geometry that reliably fits the trustworthy measurements, it produces a lower ${\mathrm{RSS}}_{m}$ for the correct cavity family than a non-robust fit would. The per-point weights $\phi_{\delta_{e},i}$ at convergence are reported in Section 3.3. After estimating θ for each cavity family m∈M, the Bayesian Information Criterion (BIC) [26] selects the most plausible model:

(11) $${\mathrm{BIC}}_{m}=n\log\left(\frac{{\mathrm{RSS}}_{m}}{n}\right)+q\log\left(n\right)+bc\left({\hat{\theta }}_{m}\right),$$

where RSS denotes the residual sum of squares between measured and predicted normalized indentation ratios:

(12) $${\mathrm{RSS}}_{m}=\sum_{i}^{k}{\left(\rho_{i}-{\hat{\rho }}_{i}\left({\hat{\theta }}_{m}\right)\right)}^{2},$$

the values *n* and *q* represent the number of measurements and the number of free parameters in the cavity model, respectively. The additional term $bc({\hat{\theta }}_{m})$ incorporates the boundary condition penalty evaluated at the estimated parameters, ensuring that models with non-physical parameter estimates are penalized in the selection process.

In practice, the nonlinear optimization exhibited a monotonic decrease of the objective energy followed by a plateau. This behavior indicates numerical stabilization of both the objective value and the estimated parameter vector under fixed hyperparameter settings.

The model with the lowest BIC was selected as the best explanation for the observed indentation responses at the estimation stage, providing both cavity-type classification and preliminary geometric parameter estimates prior to the correction stage.

#### 2.2.3. Correction Stage

The correction stage applies standard robust regression and inverse FEM principles to force-response reconciliation [6,16,32]. Its contribution lies not in the regression mechanism itself, but in its structured integration for axis-wise cavity geometry refinement under minimal interaction constraints. This stage refines cavity parameters by comparing reaction forces predicted by FEM simulations of the estimated cavity geometry against those of a homogeneous reference object. For each indentation location *i*, a normalized force ratio $ratio_{i}$ is computed as:

(13) $$ratio_{i}=\frac{F_{{\mathrm{SOFA}}_{i}}}{F_{\mathrm{SOFA}\_B_{i}}},$$

here $F_{{\mathrm{SOFA}}_{i}}$ is the reaction force predicted by SOFA for the estimated cavity geometry and $F_{\mathrm{SOFA}\_B_{i}}=F_{\mathrm{SOFA}\_B}\left(m_{full},\ell \right)$ for the homogeneous model $m_{full}$ at location *ℓ* corresponding to indentation *i*.

For each dominant axis, an iteratively reweighted least-squares (IRLS) procedure with Huber weights estimates a representative axis-level force response while suppressing local outliers [32]:

(14) $$\eta^{\left(t+1\right)}=\frac{\sum_{i}^{k}\omega_{cor_{i}}^{t}\log\left(ratio_{i}\right)}{\sum_{i}^{k}\omega_{cor_{i}}^{t}},\;\omega_{cor_{i}}^{t}=\frac{\delta_{c}}{\max(\delta_{c},|\log\left(ratio_{i}\right)-\eta^{\left(t\right)}|)},$$

here, *t* denotes the iteration index of the IRLS procedure, and $\eta^{\left(t\right)}$ is the current estimate of the robust mean at iteration *t*. The parameter $\delta_{c}$ is the Huber threshold controlling the transition between quadratic and linear weighting, thereby limiting the influence of outliers. The force ratio is log-transformed to convert multiplicative deviations into additive residuals, yielding a symmetric and scale-invariant representation suitable for least-squares estimation. At each iteration, samples whose deviation $|log\left(ratio_{i}\right)-\eta^{\left(t\right)}|$ exceeds $\delta_{c}$ are progressively downweighted, allowing the estimate to converge to a stable axis-level force indicator robust to local contact artifacts. However, this aggregation represents a structural limitation when one axis requires a scale factor substantially different from the others: the IRLS mechanism may reduce the weight of that axis rather than applying the needed correction. This behavior is documented in Section 3.3 for $M_{\mathrm{cuboid}}$.

After convergence, the axis-wise scale factor for axis $A_{s}\in \left\{X,Y,Z\right\}$ is defined as $scale_{A_{s}}=\exp\left(\eta^{t^{★}}\right)$ where $t^{★}$ denotes the final iteration. For each principal axis, the IRLS procedure [32] aggregates the available force ratios into a single robust mean η by downweighting measurements that deviate beyond $\delta_{c}$. This axis-level aggregation enables a direct one-step geometry update: rather than iterating over cavity geometry, a single scaling is applied per axis once η has converged.

When the preliminary estimate has overestimated certain cavity dimensions to the point where they approach the feasibility bound, the corresponding simulated force ratios on those axes remain close to one, providing negligible correction signal ($\log({\mathrm{ratio}}_{i})\approx 0$, yielding η≈0 and scale≈1). Any unresolved force discrepancy is consequently redistributed to the remaining axes whose correction signal remains active, concentrating the geometric update where the force response is most informative. This parameter redistribution is implemented explicitly in the correction stage: when one or more cavity dimensions reach the feasibility bound and their correction signal collapses, the remaining global force discrepancy is redistributed to the free axes, ensuring overall stiffness agreement with physical measurements even when the per-axis proportionality assumption is locally violated.

The correction stage assumes that cavity-induced force mismatches can be decomposed along the principal axes, allowing geometry refinement through independent axis-wise scaling within the selected cavity family.

For a spherical cavity, isotropy is enforced by averaging the three axis-wise scale factors:

(15) $$radius\leftarrow radius\cdot \frac{scale_{X}+scale_{Y}+scale_{Z}}{3},$$

for cuboid and cube cavities, dimensions are independently scaled along each principal axis:

(16) $$\left(a_{x},a_{y},a_{z}\right)\leftarrow \left(scale_{X}\cdot a_{x},scale_{Y}\cdot a_{y},scale_{Z}\cdot a_{z}\right),$$

for a pyramidal cavity, the base dimensions and height are scaled according to their corresponding axes:

(17) $$a_{x}\leftarrow scale_{X}\cdot a_{x},\;a_{y}\leftarrow scale_{Y}\cdot a_{y},\;h\leftarrow scale_{Z}\cdot h.$$

Here, radius denotes the radius of a spherical cavity, $\left(a_{x},a_{y},a_{z}\right)$ are the edge lengths of a cuboid cavity along the X,Y,Z axes, respectively, and $\left(a_{x},a_{y},h\right)$ denote the base dimensions and height of a pyramidal cavity, where $a_{x}$ and $a_{y}$ span the *X* and *Z* axes of the base respectively (the pyramid base lies in the XZ plane), and *h* is oriented along *Y*.

To prevent non-physical geometries, each per-point log-ratio $\log(ratio_{i})$ is clipped to a fixed interval before entering the IRLS aggregation, and the resulting axis-wise scales are jointly projected onto a feasibility range, ensuring that no single axis correction produces unbounded or physically implausible cavity dimensions.

The reliability of the correction stage depends on the quality of both the depth input and the simulated force response. Depth measurements are stabilized prior to correction by retaining the median of ten repeated indentations at each location, suppressing measurement variability before any value is imposed on the SOFA model. The repeatability of these measurements across all cavity types and gripper configurations is reported in Table A2 (see Appendix A.6). The standard deviations remain below 0.31 mm in all cases, confirming that the median is a stable representative value across all tested conditions. The resulting simulated force ratios therefore reflect cavity geometry rather than depth measurement noise. Residual inconsistencies in the force response, arising from local contact artifacts or proportionality breakdowns, are handled by the IRLS aggregation, which progressively downweights log-transformed force ratios deviating from the current robust mean. The converged axis-wise scale factor thus reflects a robust aggregate of the available force signals rather than any individual measurement. Residual geometric error after correction consequently reflects structural limitations of the pipeline rather than propagated measurement noise. These limitations, namely feasibility bound saturation and proportionality breakdown, are documented and reported in Section 3.3.

### 2.3. Materials

**Figure 3:**
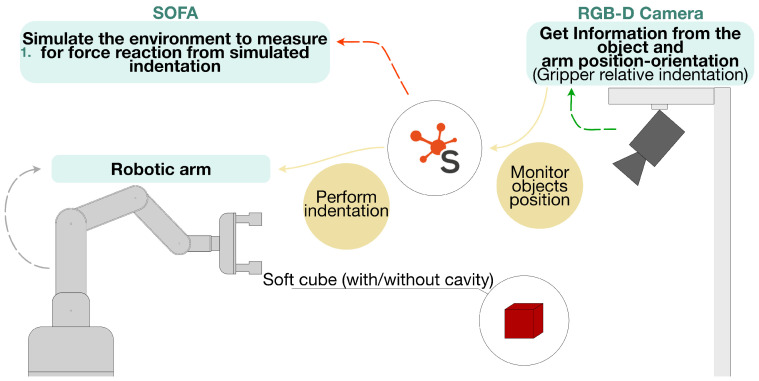
Overall project schematic.

The experimental setup consists of an myArm m750 robotic arm (Shenzhen Elephant Robotics Technology Co., Ltd., Shenzhen, China) with six degrees of freedom, equipped with a parallel-jaw gripper fabricated from PLA. The gripper behaves as a rigid body during indentation and is capable of exerting up to $F_{MAX}=7.5$ N per jaw, a value measured independently using a three-axis force sensor (USL06-H5-100N: Tec Gihan Co., Ltd., Kyoto, Japan). An D435i RGB-D camera (RealSense, Santa Clara, CA, United States) provides continuous tracking of the object’s global pose relative to the robot. ViSP (Version 3.7.0) [33] is used for markerless tracking after the camera is calibrated to the robot base. The indentation depth is computed solely from the gripper joint states and the known geometry of the cube and gripper. The camera’s role is limited to maintaining global pose alignment during indentation (Figure 3).

**Figure 4:**
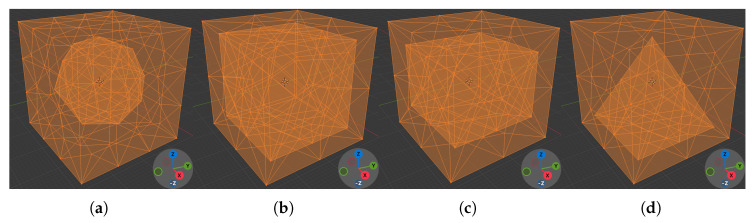
Material for the experiments: (**a**) Spherical cavity $M_{\mathrm{sphere}}$. (**b**) Cube cavity $M_{\mathrm{cube}}$. (**c**) Cuboid cavity $M_{\mathrm{cuboid}}$. (**d**) Pyramidal cavity $M_{\mathrm{pyramid}}$.

**Figure 5:**
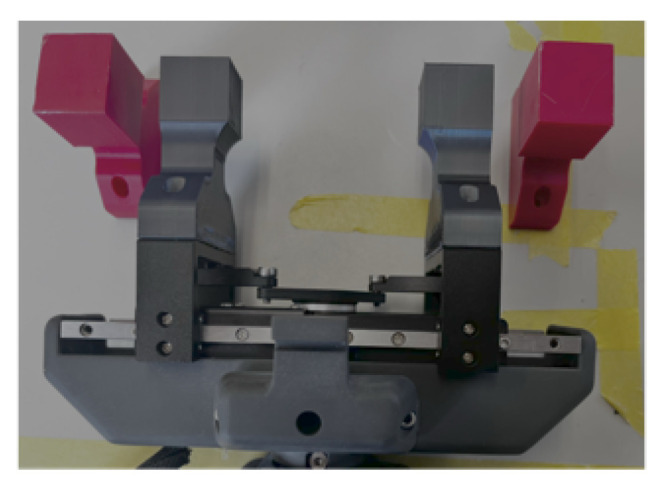
Robot gripper with 20 mm jaws $G_{20}$ (mounted), robot gripper with 25 mm jaws $G_{25}$ (unmounted).

A set of silicone cubes was fabricated using Dragon Skin 10, all of which shared identical external dimensions but differed in internal cavity geometry. Cube $M_{\mathrm{full}}$ contains no cavity. Cube $M_{\mathrm{sphere}}$ contains a spherical cavity. Cubes $M_{\mathrm{cube}}$ and $M_{\mathrm{cuboid}}$ contain cuboid cavities of different dimensions. Cube $M_{\mathrm{pyramid}}$ contains a pyramidal cavity with its apex located near the top (Figure 4). Two sets of PLA gripper jaws were printed at widths of 20 mm and 25 mm to investigate the effect of contact area (Figure 5).

The silicone mixture was degassed in a pressure chamber to minimize air bubble formation and then poured into 3D-printed molds. The mass of the cubes was measured independently. Table 1 summarizes the physical parameters of each cube, including cavity type, dimensions, material properties, and mass.

The correction stage employs a Neo-Hookean hyperelastic FEM model implemented in SOFA (Version 24.12). The Young’s modulus *E* and Poisson ratio of Dragon Skin 10 were taken from the literature [34,35,36], and a density of 1050 kg/m^3^ was used in agreement with measured values [37]. These parameters were used to compute the shear modulus, bulk modulus, and Lamé parameter as follow:

(18) $$\mu =\frac{E}{2(1+\nu )}=0.084\;\mathrm{MPa},$$

(19) $$K=\frac{E}{3(1-2\nu )}=4.17\;\mathrm{MPa},$$

(20) $$\lambda =K-\frac{2}{3}\mu =4.11\times 10^{6}\;\mathrm{Pa}.$$

which are required for the hyperelastic FEM model. For the FEM simulation, the cube was meshed using Gmsh (Version 4.15.2) [38] and discretized using linear (first-order) tetrahedral elements. Cavities were created by Boolean subtraction from a homogeneous cube, and the cavity dimensions are specified in Table 1. The resolution for each cube was generated with an element size of $h_{mesh}=30$ mm for L=50 mm chosen as a compromise between geometric accuracy and computational cost. To quantify this sensitivity, a mesh refinement test was conducted comparing $h_{mesh}=30$ mm (100 tetrahedra for $M_{\mathrm{full}}$, 167 for $M_{\mathrm{sphere}}$) and $h_{mesh}=20$ mm (197 and 328 tetrahedra respectively), with results reported in Table 2.

The force ratio $F_{M_{\mathrm{sphere}}}/F_{M_{\mathrm{full}}}$ used by the correction stage decreases from 1.257 to 1.116, a reduction of 11.2%, indicating that the coarse mesh overestimates the stiffness contrast between the cavity and homogeneous configurations. Since no experimental ground truth for the simulated force ratio exists, the finer mesh value (1.116) is taken as a closer approximation to the physical response, and the coarse mesh introduces a quantifiable overestimation of approximately 12.6% relative to this reference. Finer meshes were found to be computationally intractable for cavity geometries in SOFA.

Previous studies on silicone rubber sliding contacts report a non-constant friction coefficient that decreases with increasing nominal contact pressure [39,40]. Since SOFA uses a constant Coulomb friction model, $\mu_{f}$ was treated as a numerical parameter. A value of $\mu_{f}=0.6$ was selected consistent with the range reported for silicone rubber on polymer substrates [39,41]. In the FEM simulations, no kinematic boundary conditions were imposed on the cube; instead, gravity was enabled and a rigid horizontal plane was added to model the supporting surface. As a result, the cube’s pose and deformation evolved solely from the combination of self-weight and gripper contact forces. As a homogeneous baseline, the maximum indentation was estimated theoretically using the ideal half-space solution for a rigid flat punch [42], which provides the maximum theoretical indentation for each gripper $gr\in \{G_{20},G_{25}\}$ under the maximum applied force:

(21) $$\delta_{s_{gr}}=\frac{2(1-\nu^{2})}{\pi E}\cdot \frac{F_{MAX}}{a_{c}},\;A_{c}=s_{gr}^{2}=\pi a_{c}^{2}\Rightarrow a_{c}=\frac{s_{gr}}{\sqrt{\pi }}.$$

where $s_{gr_{i}}$ is the side length of the gripper jaw $gr_{i}$ yielding $\delta_{s_{G_{20}}}\approx 1.3$ mm and for $\delta_{s_{G_{25}}}\approx 1.0$ mm, $a_{c}$ is the radius of the equivalent circular contact area, $A_{c}$ is the contact area, and *E* and ν are the Young’s modulus and Poisson ratio of the material. To describe the contact inside SOFA, the “FrictionContactConstraint” was used for the “CollisionResponse” with the “genericConstraintSolver” and an outside QPSolver (ikine_QP from [43], Version 1.1.0) for the inverse solver [7].

**Figure 6:**
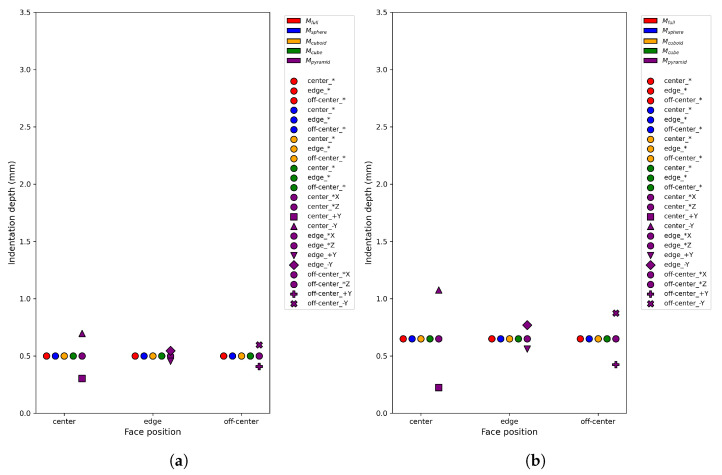
Simulated indentation in SOFA for gripper $G_{25}$ (**a**) and gripper $G_{20}$ (**b**). Predicted indentation depths for different cavity hypotheses evaluated at three face positions (center, edge, and off-center). Colors denote cavity types and marker shapes represent geometric placement variants. An asterisk (*) before an axis label indicates that both orientations of that axis (e.g., +X and −X) yield equal values, while a standalone * means all faces share the same value; the absence of * denotes location-dependent indentation with explicit signs identifying the specific face orientation.

Although the flat-punch elastic half-space model provides a reference scale for the maximum indentation under a given load, it assumes homogeneous compliance and therefore captures only the global indentation magnitude. It does not account for cavity-induced spatial redistribution of deformation. In asymmetric geometries such as pyramidal cavities, the overall indentation depth may remain close to the flat-punch reference, while the local indentation pattern varies depending on the cavity position relative to the gripper. For instance, regions closer to the cavity base exhibit deeper local indentation, whereas opposite regions remain stiffer. Such asymmetric deformation cannot be represented analytically and therefore requires FEM simulation. Accordingly, the cavity-specific indentation and reaction force responses used in the generalized error computation are obtained from SOFA. Figure 6 shows the normalized FEM-predicted indentation depths under identical loading conditions.

**Figure 7:**
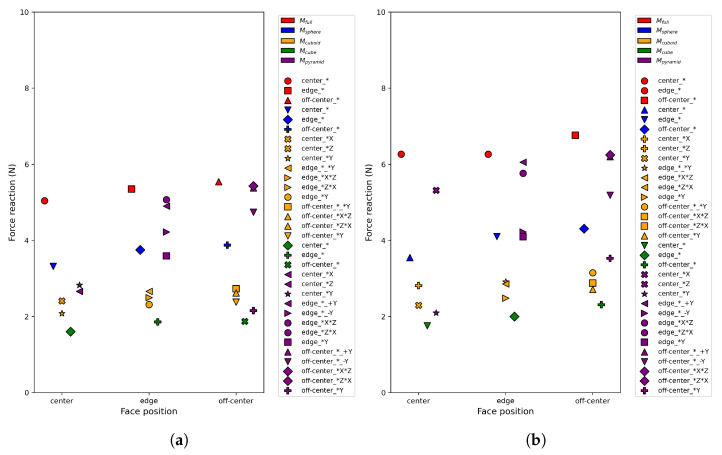
Force reaction in SOFA for gripper $G_{25}$ (**a**), and for gripper $G_{20}$ (**b**). Reaction forces predicted for different cavity hypotheses evaluated at three face positions (center, edge, and off-center). Colors denote cavity types and marker shapes represent geometric placement variants. An asterisk (*) before an axis label indicates that both orientations of that axis (e.g., +X and −X) yield equal values, while a standalone * means all faces share the same value; the absence of * denotes location-dependent indentation with explicit signs identifying the specific face orientation.

Unlike indentation magnitude, which remains bounded by the flat-punch reference scale, the reaction force predicted by FEM simulations exhibits stronger sensitivity to internal cavity geometry (Figure 7). This force variation is not merely observational; it constitutes the dominant term in the generalized error defined in Equation (1) and directly drives the margin-based discrimination criterion used to identify the minimal informative indentation set. Consequently, cavity separability in this study is primarily governed by FEM-derived force-response differences rather than indentation depth alone.

Because cavity discrimination relies on relative force-response deviations rather than absolute force matching, moderate modeling inaccuracies in FEM do not invalidate the selection or identification stages, but primarily affect the quality of geometric refinement.

### 2.4. Experimental Protocol

**Figure 8:**
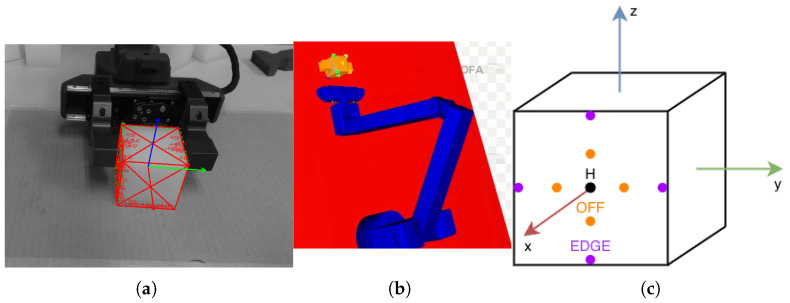
Real robot grasping cube with cube’s pose tracking (**a**), SOFA simulating grasping cube motion (**b**), and cube indentation: center (black dot), off-center as off (yellow dots), and edge (purple dots) (**c**).

The experimental validation was designed to evaluate both the identification of the minimal indentation set and the ability of the estimation–correction pipeline to reconstruct internal cavities from sparse data. Additionally, a denser set of indentation locations was used as a reference to evaluate the benefits of the minimal set. The indentations were performed along different regions of the cube surface: center, edge, and off-center locations (Figure 8c). The off-center position was defined geometrically as:

(22) $$\mathrm{off}=\frac{1}{2}\left(\frac{\mathrm{cube}\_\mathrm{size}}{2}-\frac{\mathrm{gripper}\_\mathrm{width}}{2}\right),$$

and edge positions were defined as:

(23) $$\mathrm{edge}=\frac{\mathrm{cube}\_\mathrm{size}}{2}-\frac{\mathrm{gripper}\_\mathrm{width}}{2},$$

In a first step, SOFA was used to generate simulated indentation data on a dense grid of candidate locations for all baseline cavity models and for both gripper widths. From this full simulated dataset, the generalized errors were computed, and the minimal discriminative subset $S^{*}$ was determined according to the procedure mentioned above.

In the real experiments, each cube was first indented at all locations in the grid under the maximum gripper force, while the RGB-D camera monitored the pose alignment between the cube and the robot (Figure 8a). For each cube and each indentation location (center, edge, and off-center), ten repeated indentations were performed, and the median indentation depth at each location was retained for subsequent analysis. This dense indentation dataset is used solely as a reference for comparative analysis and validation. It is not used by the proposed algorithm for cavity identification or parameter estimation.

The estimation–correction method was then applied using only the measurements corresponding to the minimal set $S^{*}$. For each cube and each gripper width, the estimation stage uses the minimal-set depths to obtain an initial cavity type and geometry. A corresponding FEM mesh is then generated for the estimated cavity and imported into SOFA for simulation. The minimal-set indentations were then reproduced in SOFA in a depth-controlled manner: for each indentation location, the median indentation depth measured in the real experiment was imposed on the corresponding SOFA model. The resulting reaction forces were used in the correction stage to update the cavity parameters (Figure 8b).

**Figure A1:**
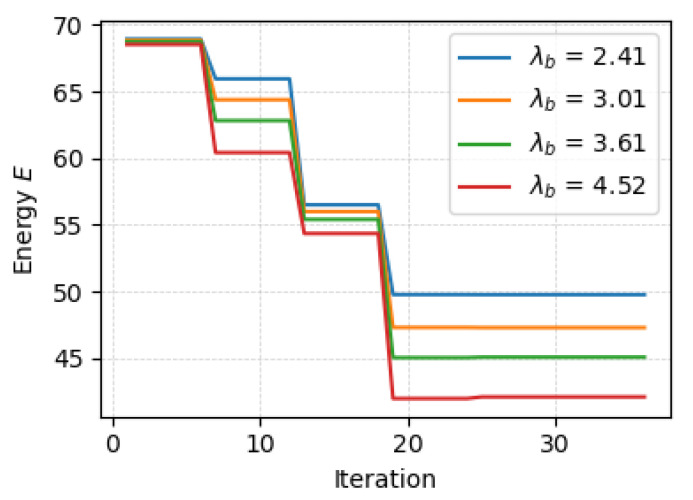
Energy evolution for different values of $\lambda_{b}$ (representative case). Across a moderate range of $\lambda_{b}$, the objective energy decreases rapidly and reaches a stable plateau, indicating practical numerical stabilization of the estimation stage under fixed initialization and solver settings.

The Huber thresholds $\delta_{e}=0.15$ and $\delta_{c}=0.30$ were set to match the expected scale of reliable residuals under the nominal experimental conditions. They were verified to produce stable cavity-type selection across the tested configurations, consistent with the robustness observed for $\lambda_{b}$ (see Appendix A.1 Figure A1).

The comparison between reconstructions obtained from the minimal set and the dense measurement set enabled a quantitative evaluation of how much information was effectively lost when restricting to $S^{*}$. In particular, classification accuracy, geometric error metrics, and the Single Reconstruction Error (SRE) were compared between the dense and minimal configurations, demonstrating that the proposed minimal set retained sufficient information for reliable cavity identification and geometry reconstruction while substantially reducing the number of required indentations.

The Single Reconstruction Error is defined as:

(24) $$SRE=\frac{RMSE}{\sqrt[{3}]{V_{True}}}\cdot 100,$$

where RMSE is the root mean square error between the estimated and true cavity geometry, and $V_{True}$ is the volume of the true cavity, whose cube root provides a characteristic length scale for normalization. This scale-invariant metric allows direct comparison of reconstruction accuracy across cavity types of different sizes.

## 3. Results

Across all experiments, the proposed framework was evaluated using two gripper configurations, $G_{25}$ and $G_{20}$, and five internal configurations: a homogeneous cube ($M_{\mathrm{full}}$) and four cavity geometries, namely spherical ($M_{\mathrm{sphere}}$), cuboid ($M_{\mathrm{cuboid}}$ and $M_{\mathrm{cube}}$), and pyramidal ($M_{\mathrm{pyramid}}$). For each object–gripper combination, cavity-type classification and geometric reconstruction were performed using the minimal indentation set composed of three centered axis-aligned indentation pairs along the *X*, *Y*, and *Z* directions.

For each configuration, three quantities are reported. First, the cavity model selected during the estimation stage, referred to as the Selected Internal Geometry (SIG). Second, the estimated cavity dimensions were provided both before and after the FEM-based correction stage, allowing a direct comparison between depth-only estimation and force-informed refinement. Third, reconstruction accuracy was quantified using multiple error metrics, including absolute error (ABS), relative percentage error, root mean square error (RMSE), cavity volume error (*V*), and the SRE. The SRE was used here as a compact diagnostic indicator of global geometric consistency rather than as an optimization objective. High SRE values indicated structural mismatch between the estimated and true cavity geometry and were expected for sharp or anisotropic cavities under sparse probing.

In all tables, the parameters include the cavity origin position *c*, the spherical radius *r*, the half-dimensions $\left(a_{x},a_{y},a_{z}\right)$ of cuboid cavities, and the base half-dimensions $b=(a_{x},a_{y})$ and height *h* of pyramidal cavities. The corresponding ground-truth values are listed in Table 1 and are used as a reference for error computation.

### 3.1. Minimal Indentation Set Validation

The minimal informative indentation set was identified using the margin-based selection criterion described in Section 2.2.1. This selection was performed prior to any real experiment and relied exclusively on pre-simulated FEM force responses obtained under controlled indentation conditions. No real-world indentation measurements were involved at this stage. Candidate indentation locations include centered, edge, and off-center positions on opposite faces of the cube along the three principal axes.

The baseline cavity models used at this stage represent cavity families rather than specific geometric instances. They were employed solely to characterize relative force-response deviations between different internal structures under identical indentation conditions. No geometric parameters or dimensional information from these models were reused during the subsequent cavity estimation stage.

For both gripper configurations, $G_{25}$ and $G_{20}$, the set composed of three centered indentation pairs along the *X*, *Y*, and *Z* axes achieved the highest separation margin among all evaluated candidate sets of the same size (Table 3). No edge or off-center indentation locations were required to satisfy the prescribed margin criterion. This result indicates that centered probing along orthogonal directions provides sufficient discriminative information to separate all cavity families considered in this study under the generalized force-error metric.

Table 3 reports the top-ranked candidate indentation sets evaluated during the selection process, together with their corresponding separation margins. The higher margin observed for $G_{20}$ (9.52 vs. 6.53 for $G_{25}$) is consistent with the narrower jaw producing more localized contact, which amplifies sensitivity to internal geometry variations and increases inter-model force-response separability. In all cases, the reported margins are strictly positive and exceed the prescribed threshold, confirming robust discriminability between cavity families. Notably, the same centered three-axis configuration was ranked highest despite the difference in jaw width between $G_{25}$ and $G_{20}$.

**Figure 9:**
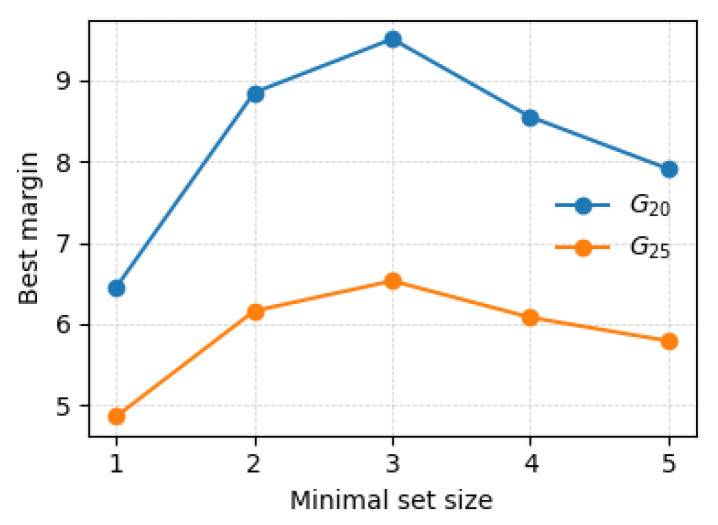
Maximum achievable separation margin as a function of indentation set size.

Figure 9 further illustrates the evolution of the maximum achievable separation margin as a function of indentation set size. The margin criterion was satisfied with three indentation groups, and the margin saturates beyond this size, indicating that additional groups yield diminishing gains in discriminability. This confirms that three groups represent the minimal sufficient set for reliable cavity discrimination in this study.

Based on these results, the three-axis centered indentation set was fixed prior to any real-world cavity estimation or FEM-based correction and used unchanged in all subsequent experiments reported in this work. The performance advantages of combining this minimal set with the proposed estimation and correction stages are evaluated in the following sections through comparison with a dense indentation baseline.

### 3.2. Cavity Estimation Validation

Using the minimal indentation set identified in the previous section, cavity-type identification and preliminary geometric estimation were performed without applying any FEM-based correction. The estimation stage relied exclusively on real-world indentation depth measurements obtained from the gripper and operated under the assumption that the internal cavity belongs to one of the predefined cavity families identified during the minimal-set discrimination stage.

**Figure 10:**
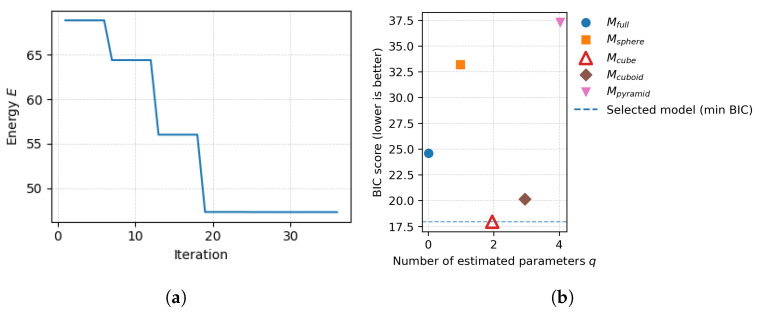
Validation of the preliminary estimation stage (estimation of $M_{\mathrm{cube}}$): Evolution of the optimization energy during parameter estimation for a representative cavity family, illustrating numerical convergence (**a**). BIC score obtained for different cavity families, plotted against the number of free parameters *k* of each model, for gripper $G_{20}$, used for cavity family selection after convergence (**b**).

To make the estimation process explicit, Figure 10 illustrates both the convergence behavior of the parameter estimation for a representative cavity family and the subsequent BIC-based model selection across all candidate families. To verify that this stabilization is not specific to a single hyperparameter choice, additional experiments were conducted by varying the decay parameter $\lambda_{b}$ within a moderate range around its nominal value. Across the tested range, the objective exhibited the same monotonic decrease followed by a stable plateau (see Appendix A.1 Figure A1), indicating that the estimation stage is numerically robust to moderate variations of $\lambda_{b}$.

For both gripper configurations, $G_{25}$ and $G_{20}$, the estimator correctly identified the internal cavity family for all tested objects, including the homogeneous case. Following convergence of the estimation for all candidate families (Figure 10a), the BIC-based selection consistently identified the correct cavity family (Figure 10b). This confirms that the minimal indentation set, composed of three centered indentations along the *X*, *Y*, and *Z* directions, provided sufficient information for reliable cavity-type selection across different end-effector geometries.

**Figure A2:**
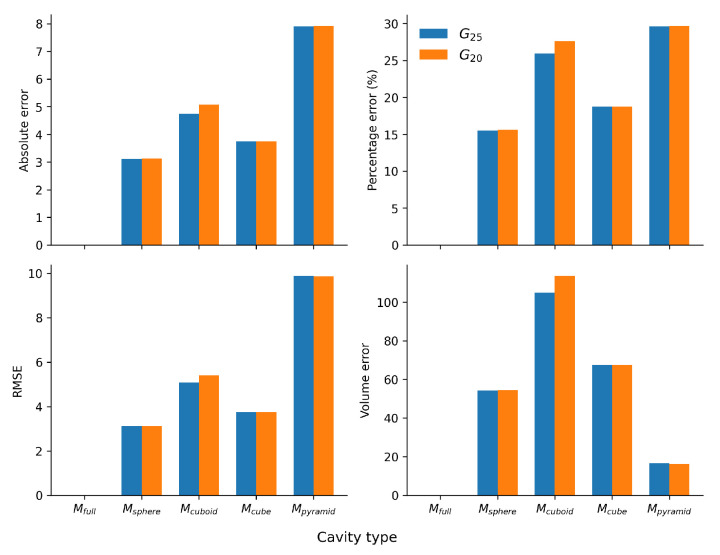
Error table of cavity-type identification and preliminary dimension estimation obtained using the minimal indentation set, before FEM-based correction.

The preliminary geometric estimates obtained for each cavity type are summarized in Table 4. For smooth and isotropic geometries, such as the spherical cavity, the estimated parameters closely match the ground truth, with percentage errors remaining below 5% for both grippers. Detailed error metrics for all cavity types before correction are provided in Figure A2 (see Appendix A.1).

In contrast, larger estimation errors were observed for cavities exhibiting sharp features or strong anisotropy, such as the cuboid and pyramidal cavities.

**Figure 11:**
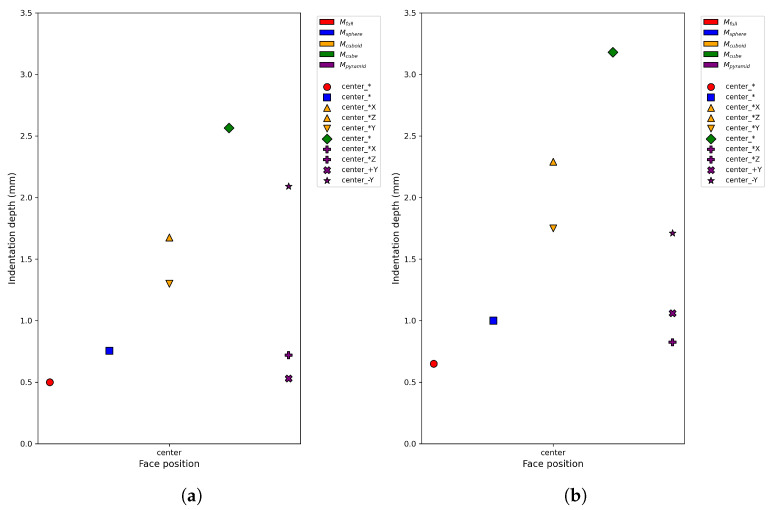
Indentation on minimal indentation set from real gripper $G_{25}$ (**a**), and gripper $G_{20}$ (**b**). Measured indentation depths for different cavity hypotheses evaluated at three face positions (center, edge, and off-center). Colors denote cavity types and marker shapes represent geometric placement variants. An asterisk (*) before an axis label indicates that both orientations of that axis (e.g., +X and −X) yield equal values, while a standalone * means all faces share the same value; the absence of * denotes location-dependent indentation with explicit signs identifying the specific face orientation.

Figure 11 illustrates the real-world indentation depths obtained using the minimal indentation set for both gripper configurations. Only the selected centered indentation locations are shown. Despite differences in absolute indentation depth induced by gripper geometry, consistent trends were observed across cavity types, supporting the robustness of the discrimination and estimation pipeline with respect to moderate variations in contact conditions.

**Figure 12:**
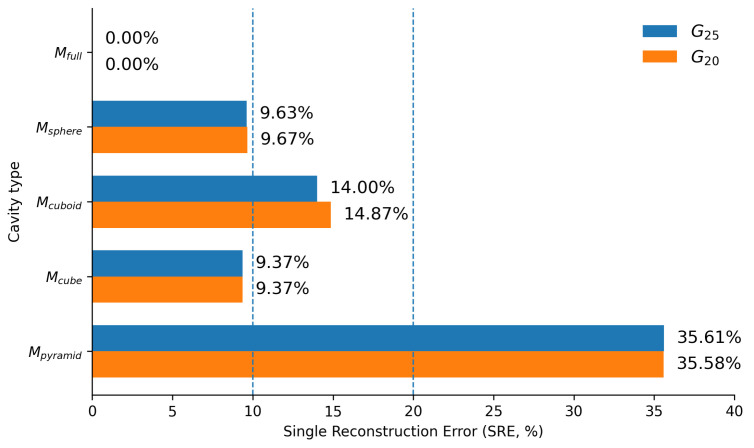
SRE cavity-type identification and preliminary dimension estimation obtained using the minimal indentation set, before FEM-based correction.

The resulting SRE values, shown in Figure 12, corroborate the detailed geometric error analysis. Low SRE values are observed for smooth and isotropic cavities, while significantly higher values are associated with sharp or anisotropic geometries.

### 3.3. Cavity Correction Validation

Following preliminary cavity identification and coarse geometric estimation, an FEM-based correction stage was applied to refine the cavity geometry using force-response consistency. In this stage, FEM was employed as a physical comparator to reconcile discrepancies between the simulated force response of the estimated cavity geometry and that of a homogeneous reference. Importantly, the correction stage operated on a fixed cavity family, as determined during the discrimination phase, and did not alter cavity-type selection.

The correction process used the same minimal indentation set identified previously. For each candidate geometry, the measured indentation depths were replayed in SOFA to obtain simulated force reactions, and geometric parameters were updated once based on the discrepancy between the simulated force response and the homogeneous reference. This procedure preserves the causal separation between discrimination, estimation, and correction by restricting FEM usage to parameter refinement within a known cavity family.

The force-based aggregation employed an IRLS formulation with Huber weights to ensure robustness to local contact artifacts and measurement noise. The IRLS formulation is employed here as described in Section 2.2.3, aggregating force ratios robustly across indentation locations.

Table 5 reports the per-axis estimation residuals, Huber weights, IRLS weights, and correction scale factors obtained for each cavity type and gripper configuration using the minimal indentation set, including ideal scale factors $s_{A_{s}}^{*}$ for reference.

**Figure 13:**
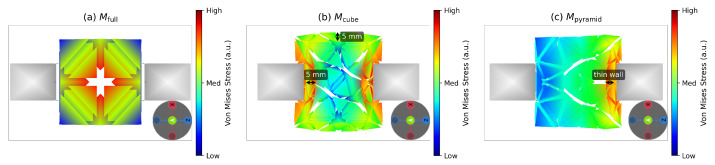
Von Mises stress distribution from SOFA under centered *Y*-axis indentation at maximum gripper force ($F_{\mathrm{MAX}}=7.5$ N) for three configurations: (**a**) homogeneous cube $M_{\mathrm{full}}$, showing a smooth stress gradient with no concentration; (**b**) cube cavity $M_{\mathrm{cube}}$, where the 40 mm cavity leaves a 5 mm wall on all faces, producing high-stress regions at the gripper–wall interface; (**c**) pyramidal cavity $M_{\mathrm{pyramid}}$, where the 40 mm base leaves a 5 mm lateral wall on the *X* and *Z* faces and the apex approaches the top surface, producing an asymmetric stress distribution.

The per-cavity weight distributions in Table 5 illustrate the two proportionality breakdown regimes introduced in Section 2.2.2: structural thinning for $M_{\mathrm{cube}}$, where $\phi_{\delta_{e},i}\approx 0.09$ uniformly across all axes, and directional geometry mismatch for $M_{\mathrm{pyramid}}$, where $\phi_{\delta_{e},i}$ varies by axis, consistent with the axis-dependent stress concentrations shown in Figure 13.

For $M_{\mathrm{full}}$, residuals are identically zero, yielding η=0 and scale=1 with no geometry change. For $M_{\mathrm{sphere}}$, residuals are small but non-negligible, producing a modest uniform scale of 0.95–0.97 consistent with Table 5.

For $M_{\mathrm{cuboid}}$, the force ratios on the *Y* axis deviate more strongly from the current mean η than those on *X* and *Z*, causing $\omega_{\mathrm{cor},Y}^{t}$ (Equation (14)) to be reduced (0.40 for $G_{25}$, 0.31 for $G_{20}$), which limits its influence on the converged η. Because *X* and *Z* are in closer agreement with each other, η is pulled toward their level, producing a uniform applied scale of 0.81, close to the ideal for *X* and *Z* (0.85) but above the ideal for *Y* (0.67). The *Y*-axis under-correction is a direct consequence of the IRLS mechanism treating the anomalous *Y* response as an outlier rather than as a corrective signal, reflecting a structural limitation when one axis requires a scale factor substantially different from the others.

For $M_{\mathrm{cube}}$, structural thinning causes the distance-based model to fail at all centered indentation sites (±X, ±Y, ±Z centered pairs), producing large residuals $r_{i}$ that reduce $\phi_{\delta_{e},i}$ uniformly to ≈0.09: the Huber mechanism effectively silences these contact points, preventing the thinning-induced nonlinearity from distorting the parameter estimate. The resulting force ratios remain consistent across axes, so $\omega_{\mathrm{cor},i}^{t}$ (Equation (14)) ≈0.50–0.61 and the applied scale (0.84) matches the ideal (0.84) closely.

For $M_{\mathrm{pyramid}}$, the overestimated base dimensions cause $\log({\mathrm{ratio}}_{i})\approx 0$ on the ±X and ±Z centered pairs, providing insufficient correction signal on those axes ($\eta_{X},\eta_{Z}\approx 0$, ${\mathrm{scale}}_{X},{\mathrm{scale}}_{Z}\approx 1$); simultaneously, the base dimensions approach the feasibility bound, blocking further growth. The unresolved force discrepancy is consequently redistributed to the remaining free axis (*Y*), where the applied scale ($s_{Y}=1.653$ for $G_{25}$ and $s_{Y}=1.529$ for $G_{20}$) partially recovers the ideal ($s_{Y}^{*}=1.69$ for $G_{25}$ and $s_{Y}^{*}=1.68$ for $G_{20}$), illustrating the causal parameter redistribution described in Section 2.2.3: the height axis achieves correction through robust consensus across the available force measurements, rather than through direct linear mapping of a single axis signal.

**Figure 14:**
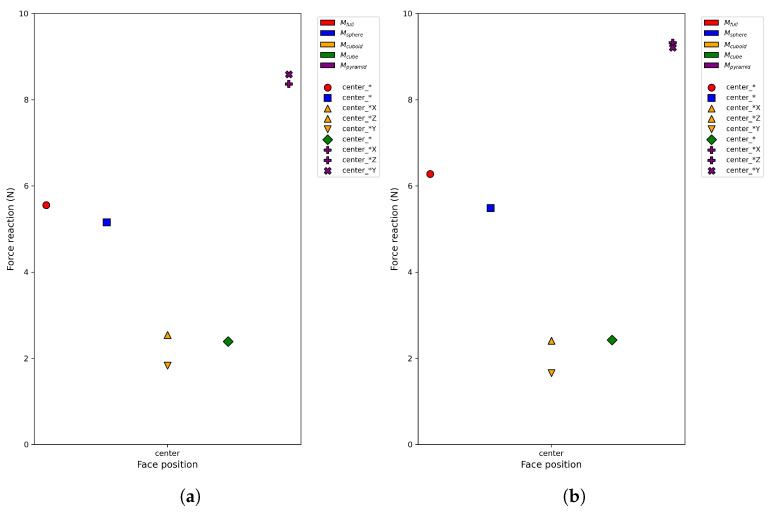
Force reaction on minimal indentation set from real gripper $G_{25}$ (**a**), and gripper $G_{20}$ (**b**). Reaction forces measured for different cavity hypotheses evaluated at three face positions (center, edge, and off-center). Colors denote cavity types and marker shapes represent geometric placement variants. An asterisk (*) before an axis label indicates that both orientations of that axis (e.g., +X and −X) yield equal values, while a standalone * means all faces share the same value; the absence of * denotes location-dependent indentation with explicit signs identifying the specific face orientation.

Figure 14a and Figure 14b illustrate the measured and FEM-simulated force-responses for grippers $G_{25}$ and $G_{20}$, respectively, after correction. For smooth and moderately anisotropic cavities, such as the spherical and cuboid cases, the corrected force-responses closely match the real measurements across all centered indentation directions.

**Figure A3:**
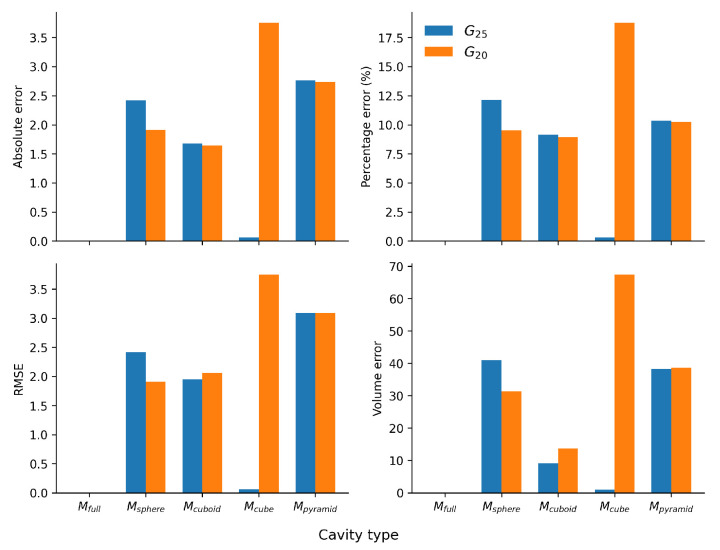
Error table of cavity-type identification and dimension estimation obtained using the minimal indentation set, after FEM-based correction.

Quantitative geometric error metrics after correction are summarized in Figure A3 (see Appendix A.2), showing reductions in ABS, RMSE, and percentage error across all non-homogeneous cavities compared to the preliminary estimation stage. The most pronounced improvements occur for geometries with directional stiffness variations, confirming that force-based correction provides information that is not accessible through depth-only probing.

Despite these improvements, residual errors remain for cavities with sharp features or strong depth-dependent stiffness gradients, particularly in the pyramidal cavity case.

**Figure 15:**
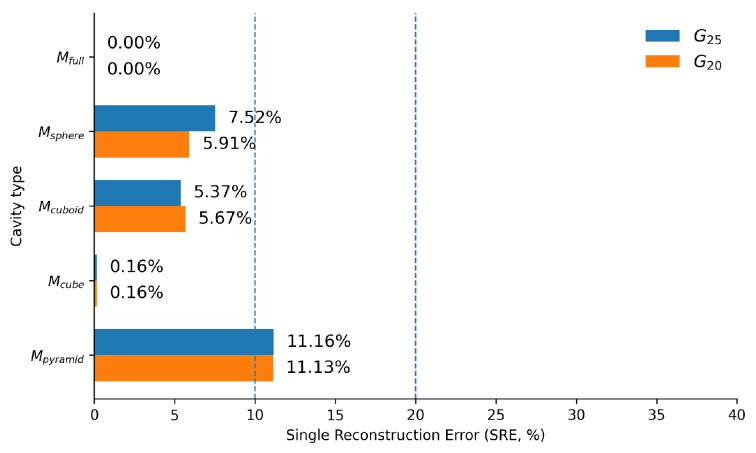
SRE cavity-type identification and dimension estimation obtained using the minimal indentation set, after FEM-based correction.

To provide a compact global assessment of correction effectiveness, the SRE was recomputed after FEM-based correction. The corrected SRE values are shown in Figure 15, with values ranging from 0.16% for $M_{\mathrm{cube}}$ to 11.16% for $M_{\mathrm{pyramid}}$. For all cavity types, the SRE decreases compared to the preliminary estimation stage, with the largest remaining error observed for the pyramidal cavity.

Table 6 summarizes the cavity-type identification and corrected geometric parameters obtained after FEM-based force reconciliation using the minimal indentation set.

The corrected estimates remain consistent across gripper configurations and preserve the cavity-type selection obtained during the discrimination stage.

The center offset $c_{y}$ of the pyramidal cavity was estimated at −13.14 mm ($G_{25}$) and −9.64 mm ($G_{20}$) against a ground truth of −20 mm (errors of 34% and 52%), as centered orthogonal indentations excite symmetric deformation modes and are thus insensitive to vertical base displacement.

### 3.4. Comparison with Full Set

**Figure A4:**
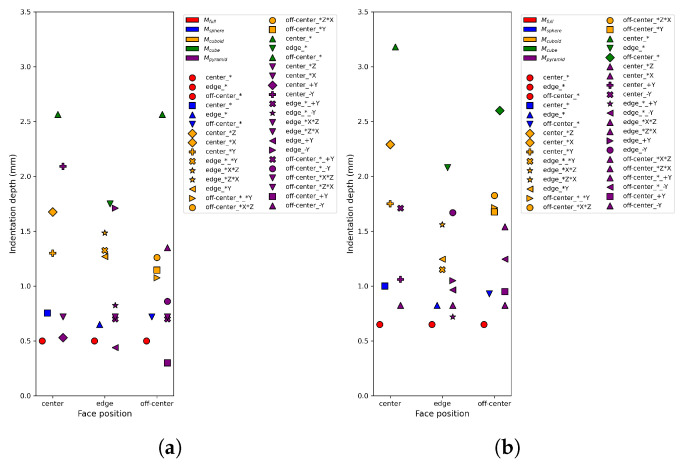
Indentation from real gripper $G_{25}$ (**a**), and gripper $G_{20}$ (**b**). Measured indentation depths for different cavity hypotheses evaluated at three face positions (center, edge, and off-center). Colors denote cavity types and marker shapes represent geometric placement variants. An asterisk (*) before an axis label indicates that both orientations of that axis (e.g., +X and −X) yield equal values, while a standalone * means all faces share the same value; the absence of * denotes location-dependent indentation with explicit signs identifying the specific face orientation.

**Figure A5:**
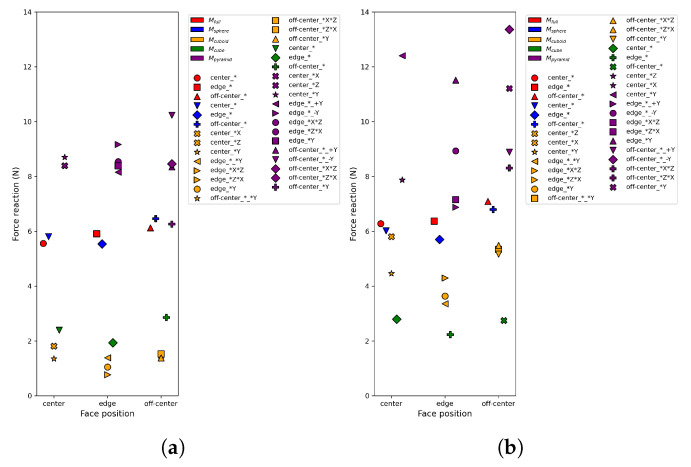
Force reaction on full indentation set from real gripper $G_{25}$ (**a**), and gripper $G_{20}$ (**b**). Reaction forces measured for different cavity hypotheses evaluated at three face positions (center, edge, and off-center). Colors denote cavity types and marker shapes represent geometric placement variants. An asterisk (*) before an axis label indicates that both orientations of that axis (e.g., +X and −X) yield equal values, while a standalone * means all faces share the same value; the absence of * denotes location-dependent indentation with explicit signs identifying the specific face orientation.

To assess both reconstruction accuracy and decision efficiency, the estimation–correction pipeline was also applied using the full indentation set, which includes centered, edge, and off-center probing locations. The objective of this comparison is not to establish an upper bound on achievable reconstruction accuracy, but to quantify the trade-off between additional physical interactions and the resulting gains in reconstruction consistency and computational cost. The full indentation set therefore serves as a reference for evaluating the marginal benefit of increased spatial sampling relative to the proposed minimal set (see data in Appendix A.3 Figure A4 and Figure A5).

**Figure 16:**
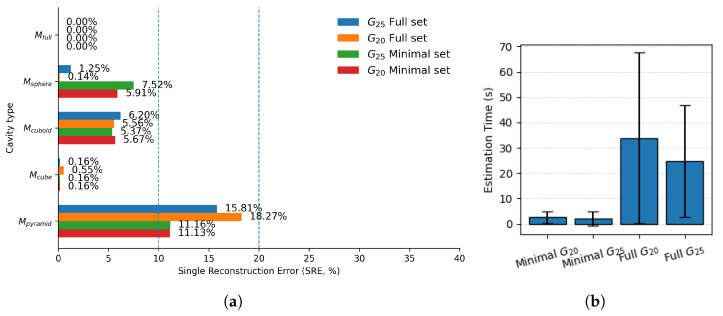
SRE cavity-type identification and dimension estimation obtained using the full indentation set, after FEM-based correction (**a**). Estimation time comparison (**b**).

**Figure A6:**
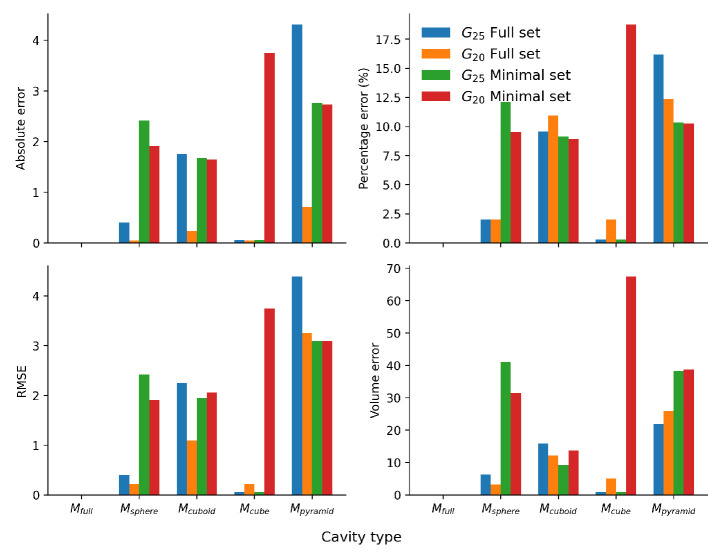
Error table of cavity-type identification and dimension estimation obtained using the full indentation set, after FEM-based correction.

Figure 16 compares both reconstruction accuracy and decision efficiency after FEM-based correction for the minimal and full indentation sets. Figure 16a reports the SRE, while Figure 16b reports the estimation time measured on the recorded indentation data. For smooth and moderately anisotropic cavities, including the spherical and cuboid cases, the full set provides only a limited reduction in SRE compared to the minimal set. In these cases, the minimal indentation strategy already achieves strong geometric consistency, indicating that additional probing locations contribute little additional corrective information. Full error metrics comparing minimal and dense indentation sets after correction are provided in Figure A6 (see Appendix A.4), showing that the minimal set achieves comparable or better performance across most metrics, with the full set providing modest additional refinement for pyramidal cavity volume estimation.

To quantify decision efficiency, Table 7 reports the computational time required to identify the cavity type and estimate preliminary geometric parameters starting from the recorded indentation measurements. Reported times include cavity-family selection and parameter estimation, but exclude robot execution and FEM simulation, which are not part of the computational pipeline. The results show that the minimal indentation set leads to a substantial reduction in decision time compared to the full set, with estimation times reduced by more than an order of magnitude. This reduction directly reflects the smaller number of indentation measurements and the reduced dimensionality of the estimation problem, without compromising cavity-type identification.

Across ABS, RMSE, and percentage error metrics, the minimal indentation set achieves performance comparable to or better than the full indentation set, even for anisotropic pyramidal cavities (See Appendix A.4 Figure A6).

Table 8 summarizes the corrected geometric parameters obtained using the full indentation set for both gripper configurations. The estimated dimensions remain physically plausible and consistent across grippers, confirming that the correction pipeline behaves robustly under increased spatial sampling. Importantly, the cavity-type selection remains unchanged, demonstrating that the use of the full set does not alter the structure of the discrimination–estimation–correction process.

## 4. Discussion

The results confirm that the three stages of the proposed framework each contribute complementary and non-redundant information—FEM-based selection, depth-driven estimation, and force-driven correction address—successive aspects of the identification problem that no single stage could resolve alone.

In particular, this study demonstrates that internal cavity discrimination can be achieved using a highly sparse and structured interaction strategy. A minimal set of three centered indentations applied along orthogonal axes was sufficient to separate all cavity types considered, as confirmed by separation margins of 6.53 and 9.52 for $G_{25}$ and $G_{20}$ respectively (Table 3), indicating that global stiffness variations induced by internal structures dominate the discriminative response.

Centered indentations reduce boundary and edge effects, producing stable force-responses that primarily reflect internal mechanical properties. When applied along orthogonal directions, these probes excite independent deformation modes, allowing cavity-induced stiffness differences to be detected without dense spatial sampling. In contrast, edge and off-center indentations mainly introduce localized effects that increase spatial resolution but do not significantly improve inter-class separability under the generalized error metric. This explains why such locations were not selected by the margin-based criterion.

The present framework was validated on Dragon Skin 10, whose mechanical properties are well characterized in the literature. Extension to objects of unknown material would require a preliminary identification step, for instance through a reference indentation on a homogeneous sample of the same material, before applying the cavity discrimination pipeline.

Finite Element Modeling plays a constrained and well-defined role in the proposed pipeline. In the selection stage, FEM-derived force responses provide the discriminative signal used to identify informative indentation locations, based exclusively on pre-simulated data and independently of any real measurements. In the correction stage, FEM refines geometric parameters only after cavity-type identification, enforcing physical consistency between the estimated cavity geometry and the measured indentation conditions. Cavity-type selection itself relies on the BIC applied to depth-only measurements, ensuring a clear separation between discrimination and refinement, as reflected in the stable cavity-type selection observed across all configurations (Table 4 and Table 6).

The correction stage assumes a local proportionality between geometric scaling and the log-transformed force ratio. While this relationship may become non-linear under extreme deformation or when the cavity boundary is in immediate proximity to the contact surface, the Huber loss in the estimation stage and the IRLS formulation in the correction stage provide complementary robustness mechanisms that detect and compensate for local proportionality breakdowns, as evidenced by the weight distributions reported in Table 5.

By utilizing Huber-weighted IRLS, the framework does not require the proportionality assumption to hold globally. When a sharp feature or structural thinning causes a non-linear force response that violates proportionality, the resulting large residual triggers a reduction in that measurement’s Huber weight, as evidenced by weight values as low as 0.09 observed for the cube cavity in Table 5. This silencing mechanism ensures that the final geometric scaling is driven only by stable, proportionate contact points, maintaining the reliability of the correction stage even in physically complex cases. However, this same mechanism represents a structural limitation when one axis requires a scale factor substantially different from the others: the IRLS aggregation may reduce the weight of that axis rather than applying the needed correction, as observed for the *Y*-axis of $M_{\mathrm{cuboid}}$ in Section 3.3. In such cases, the axis most in need of correction is precisely the one whose signal is suppressed, and the resulting under-correction is a direct consequence of the aggregation design rather than a measurement artifact.

The per-cavity manifestation of this silencing mechanism is detailed below. This is most visible for $M_{\mathrm{cube}}$, where all axes yield $\phi_{\delta_{e},i}\approx 0.09$, and for $M_{\mathrm{pyramid}}$, where $\phi_{\delta_{e},i}$ varies by axis, reflecting a directional breakdown of the proportionality assumption. The measured force response deviates most from the FEM-simulated reference; the IRLS weight $\omega_{{\mathrm{cor}}_{i}}^{t}$ is reduced accordingly, as seen for $M_{\mathrm{cuboid}}$. For $M_{\mathrm{pyramid}}$, the overestimated base dimensions produce simulated force ratios on *X* and *Z* that are close to unity, providing insufficient signal for the correction stage to reduce those dimensions. The remaining correction is therefore redistributed to the height axis ($s_{Y}=1.653$ vs. $s_{Y}^{*}=1.69$ for $G_{25}$, and $s_{Y}=1.529$ vs. $s_{Y}^{*}=1.68$ for $G_{20}$), consistent with the parameter redistribution described in Section 2.2.3. These observations confirm that the weighting mechanisms respond to genuine geometric signals and that the method remains robust even when the proportionality assumption is locally violated, within the experimental conditions of this study.

The correction stage provides the most pronounced improvements for cavities with directional stiffness variations, such as $M_{\mathrm{cuboid}}$ and $M_{\mathrm{pyramid}}$, where depth-only probing is insufficient to capture axis-dependent compliance differences. This confirms that force-based correction recovers geometric information that is not accessible through indentation depth measurements alone, and that the two stages are genuinely complementary rather than redundant.

The comparison with the full indentation set highlights a trade-off between interaction cost and geometric fidelity. Across ABS, RMSE, and percentage error metrics, the minimal set achieves reconstruction accuracy comparable to or exceeding that of the full set, even for anisotropic pyramidal cavities (See Appendix A.4 Figure A6). This indicates that the dominant stiffness characteristics governing cavity identification are captured by the centered orthogonal probes. However, for volume estimation of sharply featured geometries, the full indentation set provides additional refinement, reducing volumetric error by leveraging sensitivity to localized stiffness variations. Thus, while dense spatial probing can improve volumetric fidelity, the minimal set retains correct cavity-type identification and substantial reconstruction accuracy under a sparse interaction constraint, with estimation times reduced by more than an order of magnitude (Table 7). In the current state, the framework does not target real-time operation; the selection stage is performed prior to any real experiment, and the correction stage reduces to a closed-form per-axis computation once IRLS has converged.

Based on these results, a practical guideline can be stated. The minimal indentation set is sufficient when the cavity belongs to a known family, the material properties are known, and the internal structure is approximately centered. Under these conditions, three centered orthogonal indentations provide reliable cavity-type identification and geometric reconstruction across smooth and moderately anisotropic geometries. Denser probing does not consistently improve geometric parameter estimation and does not resolve the fundamental limitation of force and depth sensing for strongly asymmetric or significantly offset cavities, for which integration of dense surface deformation data from the RGB-D camera already present in the setup should be considered.

The residual errors observed for the pyramidal cavity, where the SRE remains above 11% even after correction (Figure 15), highlight a fundamental limitation of force and depth-only sensing: sharp geometric features produce deformation signatures insufficiently captured by scalar measurements alone. A natural extension is to exploit the RGB-D camera already present in the setup beyond global pose alignment, using dense surface deformation fields during indentation to constrain the estimation stage. Combined with FEM-based inverse analysis, this would enable finer geometric discrimination for asymmetric cavities without increasing the number of physical interactions.

### Limitations

The proposed framework operates under several assumptions that define its scope. The cavity family must be known in advance, as discrimination and estimation rely on a fixed hypothesis space. Material properties are assumed known and used directly in FEM simulations while unknown materials would require a preliminary characterization step. The method assumes approximately centered internal structures, as illustrated by the elevated reconstruction errors for the pyramidal cavity whose base origin is offset at (0,−20,0) mm. More broadly, three centered indentations are not sufficient for accurate reconstruction of sharp and strongly asymmetric cavities: the SRE remains above 11% (11.13% for gripper $G_{20}$ and 11.16% for $G_{25}$) after correction for the pyramidal cavity, reflecting a fundamental limitation of scalar force and depth measurements under sparse probing rather than a failure of the pipeline to converge. Probing at non-axial orientations, not explored in this study, or dense surface deformation data from the RGB-D camera would be required to resolve such geometries reliably.

Finally, validation was limited to a single object shape. The five cavity configurations were selected to span a representative range of geometric complexity: a homogeneous reference, an isotropic cavity (sphere), a symmetric anisotropic cavity (cube), an asymmetric anisotropic cavity (cuboid), and a strongly asymmetric cavity with a sharp feature (pyramid). This selection was intended to stress-test the pipeline across qualitatively distinct deformation regimes rather than to claim exhaustive coverage. Table 9 summarizes the validated scope of the framework, distinguishing conditions that were tested from those that remain to be investigated.

Generalization to broader object shapes, larger cavities, or more complex internal structures remains to be investigated. Extending the framework to new object shapes or cavity families would require regenerating the FEM baseline simulations and expanding the hypothesis space accordingly.

The RGB-D camera already present in the setup represents a concrete asset for addressing these limitations, as dense surface deformation tracking during indentation could provide additional geometric constraints beyond what sparse force and depth measurements alone can capture. These assumptions can be relaxed in a principled way. Relaxing the centered-structure assumption would involve expanding the hypothesis space to include cavity origin coordinates. Since the minimal set identification is performed prior to any real experiment, this expansion increases the pre-experiment simulation cost but does not require additional physical robot interactions. Relaxing the known-material assumption can be addressed by performing an initial reference indentation on a homogeneous region of the object to calibrate FEM material parameters before executing the cavity identification pipeline.

Additionally, the structural limitation of the IRLS aggregation, as discussed in Section 2.2.3 and documented in Section 3.3, may suppress correction on the axis most in need of geometric update, as observed for the *Y*-axis of $M_{\mathrm{cuboid}}$.

The use of linear tetrahedral elements with a near-incompressible material (ν=0.49) may introduce volumetric locking artifacts in FEM force predictions, which could contribute to residual geometric errors in the correction stage. FEM simulations used a coarse tetrahedral mesh ($h_{mesh}=30$ mm for a 50 mm cube) as a compromise between computational cost and accuracy. The discrimination criterion partially mitigates systematic mesh-induced errors, since it relies on relative force deviations across cavity models simulated under identical mesh conditions. However, mesh resolution still affects how accurately each cavity geometry is represented in simulation, and the reliability of the selection stage is therefore dependent on sufficient mesh resolution. The estimation stage is independent of FEM, operating on real indentation depth measurements only. The correction stage is most directly sensitive to mesh quality; a mesh sensitivity analysis confirming a 11.2% decrease in force ratio between the two mesh levels is reported in Table 2. While increasing the mesh density would improve the accuracy of FEM force predictions, the ultimate reconstruction accuracy for complex geometries such as the pyramid is also governed by the structural limitations of the axis-wise correction mechanism discussed in Section 3.3.

## 5. Conclusions

This work proposed a physics-based framework for internal cavity discrimination using a minimal number of robotic indentations. By combining margin-based selection with FEM-derived generalized force-responses, the method identifies a compact and mechanically informative interaction set prior to real-world measurements. Experimental results demonstrate that three centered, orthogonal indentations are sufficient to discriminate multiple cavity types and to enable consistent cavity-type classification across different gripper configurations. These results were obtained in a controlled setting, with known cavity families, known material properties, and approximately centered internal structures. The reported performance should therefore be interpreted within this scope, and generalization to broader conditions remains to be validated.

Across reconstruction metrics such as SRE, RMSE, and percentage error, the minimal indentation set achieves performance comparable to, and in several cases better than, the full indentation set, while reducing estimation time by more than an order of magnitude. FEM-based correction further improves geometric consistency without affecting cavity-type selection, confirming the separation between discrimination and refinement stages. For sharply featured geometries such as the pyramidal cavity, three centered indentations are not sufficient for accurate reconstruction: the SRE remains above 11% after correction, and the cavity origin along the height axis is estimated with errors of 34–52%. Dense probing provides only marginal additional volumetric refinement in this case, and resolving such geometries reliably would require probing at non-axial orientations or dense surface deformation data from the RGB-D camera already present in the setup.

By minimizing interaction complexity while preserving physical interpretability, the proposed approach supports efficient robotic decision-making under constrained sensing, within the validated scope of known cavity families, known material parameters, and approximately centered structures on a single object shape. Future work will investigate the integration of analytical cavity formulations with robotic indentation responses to establish reduced-order mechanical models, and the extension of the framework to broader cavity classes and unknown material properties.

## Figures and Tables

**Table 1:** Table of the cubes’ physical parameters (radius *r*, base *b*, height *h*). The pyramidal cavity origin is defined at the center of its base, located 20 mm below the cube center, consistent with the parameterization used in the estimation stage (Section 2.2.2).

Cube Type	Side Dimension (mm)	Cavity Type	Cavity Dimension (mm)	Cavity Origin (x,y,z) (mm)	Young’s Modulus (MPa)	Poisson Ratio	Mass (g)
$M_{\mathrm{full}}$	50	None	None	(0,0,0)	$2.5\times 10^{-1}$	0.49	130
$M_{\mathrm{sphere}}$	50	Spherical	r:20	(0,0,0)	$2.5\times 10^{-1}$	0.49	100
$M_{\mathrm{cuboid}}$	50	Cuboid	40×30×40	(0,0,0)	$2.5\times 10^{-1}$	0.49	82
$M_{\mathrm{cube}}$	50	Cube	40×40×40	(0,0,0)	$2.5\times 10^{-1}$	0.49	65
$M_{\mathrm{pyramid}}$	50	Pyramidal	b:40×40 h:40	(0,−20,0)	$2.5\times 10^{-1}$	0.49	111

**Table 2:** Mesh sensitivity analysis comparing two mesh densities for the homogeneous cube ($M_{\mathrm{full}}$) and spherical cavity ($M_{\mathrm{sphere}}$) configurations, under a fixed imposed indentation depth at the centered *Y*-axis location using gripper $G_{25}$. $h_{mesh}$: tetrahedral element size; $N_{\mathrm{tet}}$: number of tetrahedral elements; $F_{M_{\mathrm{full}}}$ and $F_{M_{\mathrm{sphere}}}$: simulated reaction forces (N) for the homogeneous and cavity configurations respectively; force ratio: $F_{M_{\mathrm{sphere}}}/F_{M_{\mathrm{full}}}$, used as the correction signal in the correction stage.

$h_{\mathrm{mesh}}$ (mm)	$N_{\mathrm{tet}}$ ($M_{\mathrm{full}}$)	$N_{\mathrm{tet}}$ ($M_{\mathrm{sphere}}$)	$F_{M_{\mathrm{full}}}$ (N)	$F_{M_{\mathrm{sphere}}}$ (N)	Force Ratio
30	100	167	9.22	11.59	1.257
20	197	328	8.26	9.22	1.116

**Table 3:** Top-ranked indentation sets evaluated by the margin-based selection procedure for both gripper configurations.

Gripper	Selected Indentation Pairs	Indentation Locations	Margin	Set Size	Rank
$G_{25}$	Centered *X*, *Y*, *Z* axis	$\pm X_{c},\;\pm Y_{c},\;\pm Z_{c}$	6.53	3	1
$G_{25}$	Centered *X*, *Y* axis	$\pm X_{c},\;\pm Y_{c}$	6.15	2	2
$G_{25}$	Centered *Y*, *Z* axis	$\pm Y_{c},\;\pm Z_{c}$	6.15	2	2
$G_{25}$	Centered *X*, *Z* axis, *X*+*Z* off	$\pm X_{c},\;\pm Z_{c},\;\pm X$+$Z_{off}$	5.65	3	3
$G_{25}$	Centered *X*, *Z* axis, *X*-*Z* off	$\pm X_{c},\;\pm Z_{c},\;\pm Z$-$Y_{off}$	5.65	3	3
$G_{20}$	Centered *X*, *Y*, *Z* axis	$\pm X_{c},\;\pm Y_{c},\;\pm Z_{c}$	9.52	3	1
$G_{20}$	Centered *X*, *Y* axis	$\pm X_{c},\;\pm Y_{c}$	8.85	2	2
$G_{20}$	Centered *Y*, *Z* axis	$\pm Y_{c},\;\pm Z_{c}$	8.85	2	2
$G_{20}$	Centered *X*, *Z* axis, *X*+*Z* edge	$\pm X_{c},\;\pm Z_{c},\;\pm X$+$Z_{edge}$	7.68	3	3
$G_{20}$	Centered *Y*, *Z* axis, *X*+*Y* edge	$\pm Y_{c},\;\pm Z_{c},\;\pm X$+$Y_{edge}$	7.68	3	3

**Table 4:** Cavity-type identification and preliminary dimension estimation obtained using the minimal indentation set, before FEM-based correction. Dimensions are reported as full edge lengths, equal to twice the half-length parameters used internally in the estimation stage.

	Minimal Set $G_{25}$		Minimal Set $G_{20}$	
Cavity	SIG	Dimension (mm)	SIG	Dimension (mm)
Full	$M_{\mathrm{full}}$	None	$M_{\mathrm{full}}$	None
Spherical	$M_{\mathrm{sphere}}$	c:(0,0,0)	$M_{\mathrm{sphere}}$	c:(0,0,0)
		r:23.11		r:23.12
Cuboid	$M_{\mathrm{cuboid}}$	c:(0,0,0)	$M_{\mathrm{cuboid}}$	c:(0,0,0)
		46.95×44.63×46.95		47.5×45.42×47.5
Cube	$M_{\mathrm{cube}}$	c:(0,0,0)	$M_{\mathrm{cube}}$	c:(0,0,0)
		47.5×47.5×47.5		47.5×47.5×47.5
Pyramid	$M_{\mathrm{pyramid}}$	c:(0,−13.14,0)	$M_{\mathrm{pyramid}}$	c:(0,−9.64,0)
		b:47.4×47.4		b:47.5×47.5
		h:23.72		h:23.75

**Table 5:** Per-axis estimation residuals $\left(\rho_{i}-{\hat{\rho }}_{i}\right)$, Huber weights $\phi_{\delta_{e},i}$, IRLS weights $\omega_{{\mathrm{cor}}_{i}}^{t}$, applied scale factors $s_{A}$, and ideal scale factors $s_{A}^{*}$ (the ratio of ground-truth to estimated cavity dimension along axis *A*, shown in parentheses) for each cavity type and gripper. Large residual magnitudes indicate local proportionality breakdowns, to which $\phi_{\delta_{e},i}$ and $\omega_{{\mathrm{cor}}_{i}}^{t}$ respond by reducing the influence of the affected measurements. For the pyramidal cavity, +Y and −Y are listed separately; all other ± pairs are collapsed (for pyramidal cavity, $s_{Y}$ rescale the height).

		Minimal Set $G_{25}$				Minimal Set $G_{20}$			
Cavity	Axis	$\rho_{i}-{\hat{\rho }}_{i}$	$\phi_{\delta_{e},i}$	$\omega_{{\mathrm{cor}}_{i}}^{t}$	Scale $s_{X}/s_{Y}/s_{Z}$ Ideal $\left(s_{X}^{*}/s_{Y}^{*}/s_{Z}^{*}\right)$	$\rho_{i}-{\hat{\rho }}_{i}$	$\phi_{\delta_{e},i}$	$\omega_{{\mathrm{cor}}_{i}}^{t}$	Scale $s_{X}/s_{Y}/s_{Z}$ Ideal $\left(s_{X}^{*}/s_{Y}^{*}/s_{Z}^{*}\right)$
Full	±X	0.00	1.00	1.00	1.00/1.00/1.00 (−−−)	0.00	1.00	1.00	1.00/1.00/1.00 (−−−)
	±Y	0.00	1.00	1.00		0.00	1.00	1.00	
	±Z	0.00	1.00	1.00		0.00	1.00	1.00	
Spherical	±X	≈0	1.00	1.00	0.97/0.97/0.97 (0.87/0.87/0.87)	≈0	1.00	1.00	0.95/0.95/0.95 (0.86/0.86/0.86)
	±Y	≈0	1.00	1.00		≈0	1.00	1.00	
	±Z	≈0	1.00	1.00		≈0	1.00	1.00	
Cuboid	±X	≈0	1.00	0.69	0.81/0.81/0.81 (0.85/0.67/0.85)	−0.03	1.00	0.49	0.81/0.81/0.81 (0.85/0.68/0.85)
	±Y	≈0	1.00	0.40		≈0	1.00	0.31	
	±Z	≈0	1.00	0.69		−0.03	1.00	0.49	
Cube	±X	−1.73	0.09	0.61	0.84/0.84/0.84 (0.84/0.84/0.84)	−1.62	0.09	0.50	0.84/0.84/0.84 (0.84/0.84/0.84)
	±Y	−1.73	0.09	0.61		−1.62	0.09	0.50	
	±Z	−1.73	0.09	0.61		−1.62	0.09	0.50	
Pyramid	±X	−0.50	0.30	1.00	1.00/1.653/1.00 (0.84/1.69/0.84)	−0.31	0.49	1.00	1.00/1.529/1.00 (0.84/1.68/0.84)
	+Y	+0.22	0.68	1.00		−0.36	0.42	1.00	
	−Y	+0.19	0.78	1.00		−0.33	0.45	1.00	
	±Z	−0.50	0.30	1.00		−0.31	0.49	1.00	

**Table 6:** Cavity-type identification and dimension estimation obtained using the minimal indentation set, after FEM-based correction. Dimensions are reported as full edge lengths, equal to twice the half-length parameters used internally in the estimation stage.

	Minimal Set $G_{25}$		Minimal Set $G_{20}$	
Cavity	SIG	Dimension (mm)	SIG	Dimension (mm)
Full	$M_{\mathrm{full}}$	None	$M_{\mathrm{full}}$	None
Spherical	$M_{\mathrm{sphere}}$	c:(0,0,0)	$M_{\mathrm{sphere}}$	c:(0,0,0)
		r:22.42		r:21.91
Cuboid	$M_{\mathrm{cuboid}}$	c:(0,0,0)	$M_{\mathrm{cuboid}}$	c:(0,0,0)
		38.06×36.18×38.06		38.5×36.82×38.5
Cube	$M_{\mathrm{cube}}$	c:(0,0,0)	$M_{\mathrm{cube}}$	c:(0,0,0)
		39.9×39.9×39.9		39.9×39.9×39.9
Pyramid	$M_{\mathrm{pyramid}}$	c:(0,−13.14,0)	$M_{\mathrm{pyramid}}$	c:(0,−9.64,0)
		b:47.5×47.5		b:47.5×47.5
		h:39.22		h:36.33

**Table 7:** Comparison of decision efficiency between the minimal indentation set and the full indentation set. Selection time for the full set is 0.0 s because no margin-based selection is performed; all locations are used directly. Correction time is 0.0 s for all configurations because the scale update reduces to a per-axis closed-form computation once IRLS has converged. $S^{*}$ denotes the minimal collection of indentation set.

Candidate Set Evaluated	Number of Indentations	Selection Time (s)	Estimation Time (s)	Correction Time (s)
Minimal $S^{*}$ $G_{25}$	6	0.25	2.58	0.0
Minimal $S^{*}$ $G_{20}$	6	0.25	1.98	0.0
Full $G_{25}$	54	0.0	33.81	0.0
Full $G_{20}$	54	0.0	24.75	0.0

**Table 8:** Cavity-type identification and dimension estimation obtained using the full indentation set, after FEM-based correction.

	Full Set $G_{25}$		Full Set $G_{20}$	
Cavity	SIG	Dimension (mm)	SIG	Dimension (mm)
Full	$M_{\mathrm{full}}$	None	$M_{\mathrm{full}}$	None
Spherical	$M_{\mathrm{sphere}}$	c:(0,0,0)	$M_{\mathrm{sphere}}$	c:(0,0,0)
		r:20.40		r:20.05
Cuboid	$M_{\mathrm{cuboid}}$	c:(0,0,0)	$M_{\mathrm{cuboid}}$	c:(0,0,0)
		38.5×37.5×38.5		35.6×33.2×35.6
Cube	$M_{\mathrm{cube}}$	c:(0,0,0)	$M_{\mathrm{cube}}$	c:(0,0,0)
		39.9×39.9×39.9		39.6×39.6×39.6
Pyramid	$M_{\mathrm{pyramid}}$	c:(0,−12.3,0)	$M_{\mathrm{pyramid}}$	c:(0,−9.07,0)
		b:47.5×47.5		b:37.0×37.0
		h:34.56		h:31.25

**Table 9:** Validated scope of the proposed framework.

Condition	Status
Known cavity families	Tested
Known material parameters	Tested
Centered internal structures	Tested (approximately)
Non-centered internal structures	Not tested (partly addressed by pyramidal cavity)
Unknown material parameters	Not tested
Non-cubic external object shape	Not tested
Cavities beyond the four tested families	Not tested

## Data Availability

The dataset supporting the findings of this study is publicly available on GitHub and archived on Zenodo at https://doi.org/10.5281/zenodo.20072729.

## Abbreviations

The following abbreviations are used in this manuscript:

FEM	Finite Element Method
PLA	Polylactide
BIC	Bayesian Information Criterion
RSS	Residual Sum of Squares
IRLS	Iteratively Reweighted Least Squares
RMSE	Root Mean Square Error
SRE	Single Reconstruction Error
ABS	Absolute Error
SIG	Selected Internal Geometry

## Appendix A

### Appendix A.5

**Table A1:** Hyperparameters of the estimation framework and their values.

Parameter	Symbol	Value	Role
Spatial decay	$\lambda_{b}$	3.0 mm	Influence decay from cavity boundary
Huber threshold	$\delta_{e}$	0.15	Robust loss transition in parameter fitting
Huber threshold	$\delta_{c}$	0.30	Huber threshold in IRLS force correction
Pairwise weight cap	$\omega_{\mathrm{cap}}$	3.0	Limits antisymmetry pair contribution
Antisymmetry scale	$\sigma_{\mathrm{anti}}$	0.12	Normalizes pairwise weighting
Pointwise reweight	$\omega_{i}$	0.30	Uniform contribution of indentation locations
Size penalty strength	β	25.0log(n)	BIC penalty for non-physical cavity sizes
Noise level	σ	0.02	Assumed measurement noise for margin criterion

### Appendix A.6

**Table A2:** Repeatability of indentation depth measurements at the minimal set locations (n=10 repetitions per location). Median, mean, and standard deviation are reported per axis direction and configuration. For $M_{\mathrm{pyramid}}$, the ±Y depths differ due to the asymmetric cavity placement. The − and + signs denote the negative and positive directions along each axis, respectively (e.g., −X and +X correspond to indentations on opposite faces along the *X* axis).

Config.	Axis	Median (mm)		Mean (mm)		Std (mm)	
		−	+	−	+	−	+
$G_{20}\;M_{\mathrm{full}}$	*X*	1.300	1.300	1.275	1.297	0.080	0.066
	*Y*	1.300	1.300	1.255	1.301	0.092	0.097
	*Z*	1.300	1.300	1.270	1.301	0.074	0.079
$G_{20}\;M_{\mathrm{sphere}}$	*X*	2.000	2.000	2.086	2.066	0.172	0.117
	*Y*	2.000	2.000	2.070	2.059	0.161	0.136
	*Z*	2.000	2.000	2.037	2.029	0.135	0.141
$G_{20}\;M_{\mathrm{cuboid}}$	*X*	4.580	4.580	4.615	4.667	0.076	0.230
	*Y*	3.500	3.500	3.660	3.550	0.246	0.138
	*Z*	4.580	4.580	4.726	4.740	0.308	0.304
$G_{20}\;M_{\mathrm{cube}}$	*X*	6.360	6.360	6.406	6.368	0.062	0.096
	*Y*	6.360	6.360	6.374	6.360	0.072	0.057
	*Z*	6.360	6.360	6.406	6.339	0.062	0.094
$G_{20}\;M_{\mathrm{pyramid}}$	*X*	1.650	1.650	1.713	1.726	0.116	0.115
	*Y*	1.710	1.060	1.723	1.069	0.025	0.017
	*Z*	1.650	1.650	1.713	1.723	0.116	0.115
$G_{25}\;M_{\mathrm{full}}$	*X*	1.000	1.000	0.990	1.001	0.059	0.047
	*Y*	1.000	1.000	0.994	0.979	0.052	0.059
	*Z*	1.000	1.000	1.016	1.005	0.034	0.049
$G_{25}\;M_{\mathrm{sphere}}$	*X*	1.510	1.510	1.516	1.517	0.013	0.022
	*Y*	1.510	1.510	1.531	1.513	0.034	0.010
	*Z*	1.510	1.510	1.519	1.520	0.015	0.023
$G_{25}\;M_{\mathrm{cuboid}}$	*X*	3.350	3.350	3.347	3.378	0.033	0.073
	*Y*	2.600	2.600	2.626	2.629	0.094	0.102
	*Z*	3.350	3.350	3.332	3.352	0.036	0.063
$G_{25}\;M_{\mathrm{cube}}$	*X*	5.130	5.130	5.169	5.123	0.071	0.040
	*Y*	5.130	5.130	5.136	5.115	0.059	0.062
	*Z*	5.130	5.130	5.136	5.129	0.059	0.055
$G_{25}\;M_{\mathrm{pyramid}}$	*X*	1.440	1.440	1.446	1.410	0.038	0.063
	*Y*	2.090	0.530	2.267	0.579	0.278	0.077
	*Z*	1.440	1.440	1.402	1.439	0.061	0.030

### Appendix A.7

**Algorithm A1:** Construction of the weighted generalized error matrix. For each cavity hypothesis and indentation location, normalized force (and optionally depth) discrepancies are combined into a generalized error E[m,ℓ]. Location-dependent weights are computed to (i) balance spatial sampling across regions and (ii) emphasize locations with higher inter-model dispersion. The resulting weighted error matrix serves as the basis for model discrimination and minimal set selection

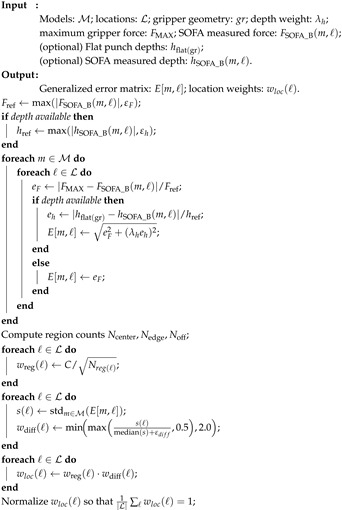

### Appendix A.8

**Algorithm A2:** Minimal informative group selection using a margin criterion. Candidate indentation groups are incrementally combined with a base set to form subsets *S*. For each subset, weighted error vectors are constructed for all cavity hypotheses, and their minimum pairwise Euclidean distance defines a separation margin. The smallest subset whose margin exceeds the threshold γ is selected as the informative set $S^{*}$; among subsets of equal minimal size, the one achieving the highest margin is retained
